# Cobalt Pyrene‐Quaterpyridine Molecular Complex Immobilized on Functionalized Multi‐Walled Carbon Nanotubes as a Robust Hybrid Catalyst for Efficient Electrochemical Reduction of CO_2_

**DOI:** 10.1002/advs.202509854

**Published:** 2025-09-16

**Authors:** Yue Wei, Wangxiyue Yi, Lingjing Chen, Huatian Shi, Marc Robert, Tai‐Chu Lau, Gui Chen

**Affiliations:** ^1^ School of Environment and Civil Engineering Research Center for Eco‐environmental Engineering Guangdong Provincial Key Laboratory of Intelligent Disaster Prevention and Emergency Technologies for Urban Lifeline Engineering Dongguan University of Technology Dongguan Guangdong 523808 P. R. China; ^2^ Sorbonne Université CNRS Institut Parisien de Chimie Moléculaire Paris F‐75005 France; ^3^ Institut Universitaire de France (IUF) Paris F‐75005 France; ^4^ Department of Chemistry City University of Hong Kong Tat Chee Avenue Kowloon Tong Hong Kong 999077 P. R. China

**Keywords:** carbon nanotubes, CO_2_ reduction, Cobalt quaterpyridine, electrocatalysis, *π–π* interactions

## Abstract

Molecular complexes immobilized on conductive carbon supports is a promising strategy for designing efficient electrocatalysts of CO_2_ reduction reaction (CO_2_RR). However, most immobilized catalysts suffer from low catalytic activity and durability due to weak catalyst‐support interactions. Herein, modifications of Co quaterpyridine complex (Coqpy) with a pyrene group and multi‐walled carbon nanotubes (MWCNT) with carboxyl or amide groups have been made, resulting in a highly efficient and stable hybrid catalyst. The carboxyl groups on MWCNT can effectively bind to Co centers by axial coordination; which allow for loading more electroactive Co complex, facilitate interfacial electron transfer, and lower the energy barrier for CO_2_RR. A 2.6‐fold increase in CO yield and a higher Faradaic efficiency (97.5% vs 83.6% at 423 mV overpotential) are obtained compared with the hybrid catalyst without carboxyl group. While, the pyrene group induced more substantial *π–π* interactions, leading to an enhanced electronic coupling between MWCNT and Coqpy, and enhanced hybrid stability, resulting in a ≈ 4.4‐fold higher CO turnover frequency than Coqpy. Overall, the outstanding performance of the modified hybrid catalyst (turnover number up to 570000, Faradaic efficiency close to 100%) provides new mechanistic insights and design strategy for efficient and durable CO_2_RR based on heterogenized molecular catalysts.

## Graphical abstract

The efficiency and durability of Coqpy@MWCNT hybrids (qpy = quaterpyridine) for electrocatalytic CO_2_ reduction are significantly improved by using coordination and enhanced *π–π* interaction strategy, with TOF of 79 s^−1^, TON of 5.7  × 10^5^, FE close to 100%; which is achieved by functionalization of MWCNT with carboxyl groups and appending a pyrene group to the qpy ligand.

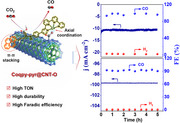

## 1 Introduction

Electrochemical carbon dioxide reduction reaction (CO_2_RR) using solar‐derived electricity to produce renewable fuels is a promising approach for a sustainable carbon‐neutral economy.^[^ ^1^, ^2^ ^]^ Despite tremendous efforts, the design of practical CO_2_RR electrocatalysts with high activity, selectivity, and stability remains a challenge to chemists.^[^ ^3^ ^]^ Molecular catalysts have been widely investigated because they lead to high activity and selectivity, and the CO_2_RR mechanism can be readily probed due to their structural tunability.^[^ ^4^, ^5^ ^]^ Molecular catalysts can now be seriously envisioned as electrocatalysts for CO_2_ emerging electrolyzers, upon anchoring them onto carbon based catalytic electrodes.^[^ ^3^, ^5^, ^6^, ^7^, ^8^, ^9^, ^10^, ^11^ ^]^

The most common approach for such strategy is through *π–π* stacking of planar aromatic/unsaturated macrocyclic ligands with sp^2^‐carbon supports.^[^ ^12^, ^13^ ^]^ However, in most cases, this interaction is relatively weak, thus limiting both the stability of the hybrid catalysts and the fraction of the deposited molecular complexes being electrochemically addressed.^[^ ^14^, ^15^, ^16^, ^17^, ^18^ ^]^ There are also a few examples of immobilization through the coordination of the metal centers to the solid supports, but these are mainly based on rather complicated designs.^[^ ^12^, ^19^, ^20^, ^21^, ^22^ ^]^ Also, the effects of coordination to solid supports on the metal electronic structure, which could influence the adsorption and activation of intermediates,^[^ ^23^, ^24^, ^25^, ^26^ ^]^ remains poorly understood. Hence, an effective immobilization strategy that would result in rapid electronic transfer between hybrid catalyst, as well as maximizing the activity and stability of the molecular catalyst are essential for achieving highly efficient CO_2_RR. Some of us have recently reported that the Co complex of the planar tetradentate ligand 2,2′:6′,2″:6″,2′″‐quaterpyridine (Coqpy) is an active catalyst for both photocatalytic^[^ ^27^ ^]^ and electrochemical^[^ ^28^ ^]^ CO_2_ reduction in aprotic solvent, with the generation of CO in high yields and selectivity. Coqpy has also been covalently linked to carbon nitride^[^ ^29^, ^30^ ^]^ and graphene,^[^ ^31^ ^]^ and the resultant hybrid materials are highly robust photocatalysts for CO_2_ reduction. Coqpy has also been immobilized on multi‐walled carbon nanotubes (MWCNT) for CO_2_‐to‐CO conversion in aqueous solution at neutral pH, with 100% selectivity and 100% Faradaic efficiency (FE).^[^ ^32^ ^]^ These studies demonstrated that Coqpy provides a promising platform for obtaining excellent CO_2_RR performance via suitable immobilization strategy.

**Figure 1:**
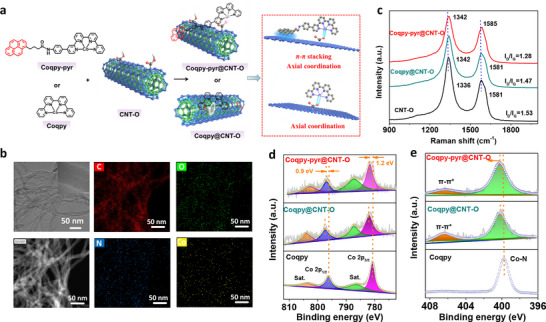
Preparation and characterization of the hybrid catalysts. a) Immobilization strategy of Coqpy/Coqpy‐pyr onto CNT‐O and interactions involved. b) HR‐TEM images, high‐angle annular dark‐field scanning transmission electron microscopy (HAADF‐STEM) image and corresponding elemental mappings of Coqpy@CNT‐O. c) Raman spectra of CNT‐O, Coqpy@CNT‐O, and Coqpy‐pyr@CNT‐O. High‐resolution (d) Co 2p and (e) N 1s XPS spectra of Coqpy, Coqpy@CNT‐O, and Coqpy‐pyr@CNT‐O.

In this work, we have modified the Coqpy molecular catalyst by appending a pyrene group to the qpy ligand (**Figure** **1**a, denoted as Coqpy‐pyr). The presence of four fused benzene rings on pyrene should significantly enhance *π–π* interactions of Coqpy‐pyr with MWCNT. In addition, we have also modified MWCNT by adding ‐COOH (denoted as CNT‐O) or ‐NH_2_ (denoted as CNT‐N) functional groups on its surface. These functional groups can coordinate to Coqpy/Coqpy‐pyr and further facilitate binding of the Co complexes to CNT‐O and CNT‐N. We report herein that Coqpy/Coqpy‐pyr@CNT‐O/CNT‐N are more efficient than the unmodified Coqpy/Coqpy‐pyr@CNT for CO_2_‐to‐CO conversion in aqueous solution. In particular, Coqpy‐pyr@CNT‐O stands among the best electrocatalysts for CO_2_RR in terms of catalytic efficiency, FE, and durability.

## 2 Results and Discussion

### 2.1 Catalyst Synthesis and Characterizations

The synthesis of Coqpy‐pyr is summarized in Scheme S1 (Supporting Information). Coqpy and Coqpy‐pyr immobilized on MWCNT functionalized with different groups (none, carboxyl or amido, abbreviated as CNT, CNT‐O, and CNT‐N, respectively) were prepared and investigated as electrocatalysts for CO_2_RR. The hybrid catalysts are denoted as Coqpy/Coqpy‐pyr@CNT, Coqpy/Coqpy‐pyr@CNT‐O, and Coqpy/Coqpy‐pyr@CNT‐N. Catalyst inks containing the Co catalysts and MWCNT were prepared in ethylene glycol/ethanol by a method similar to that previously reported for Coqpy@MWCNT,^[^ ^32^ ^]^ and were drop‐casted onto carbon fiber paper electrode (SI). Typical catalyst loadings are 8 – 27 nmol per cm^2^ of electrode. These electrodes were used for electrocatalytic CO_2_RR.

Catalyst characterizations were mainly performed with samples isolated from the catalyst inks. High‐resolution TEM (HR‐TEM) images of Coqpy@CNT‐O reveal its 1D nanotube morphology and the well‐defined inner wall of CNT‐O (Figure 1b). Co nanoparticles completely encapsulated within the compartments of CNT‐O are observed (Figure S4, Supporting Information). These nanoparticles exhibit a spacing of 2.04 Å, corresponding to the (111) plane of metallic Co (Figure S3, Supporting Information), originating from the use of Co catalyst in the preparation of CNT.^[^ ^33^ ^]^ The encapsulation of Co nanoparticles rules out their possible origin from Coqpy, since no Co particles exist on the outer shells. The energy dispersive X‐ray spectroscopy (EDS) elemental mappings further show that the N and Co atoms of Coqpy are highly distributed on the CNT‐O (Figure 1b). A similar TEM result was found for Coqpy‐pyr@CNT‐O (Figure S5, Supporting Information). In the UV‐vis spectra of Coqpy@CNT‐O and Coqpy‐pyr@CNT‐O, enhanced absorption bands are found between 200 and 350 nm compared to CNT‐O (Figure s6, Supporting Information), which can be assigned to π–π* transitions of the qpy and qpy‐pyr ligands,^[^ ^34^ ^]^ indicating the successful immobilization of the molecular catalysts on CNT‐O.

The Raman spectra of Coqpy@CNT‐O and Coqpy‐pyr@CNT‐O show characteristic peaks at around 1340 and 1580 cm^−1^ (Figure 1c), that are ascribed to the surface defect (D band) and lattice vibration of graphitic carbon (G band),^[^ ^14^ ^]^ respectively. The smaller I_D_/I_G_ values of the samples relative to pristine CNT‐O reflect the decreased degree of disorder upon binding Co complex to the sp^2^‐hybridized carbon matrix.^[^ ^35^ ^]^ Compared with Coqpy@CNT‐O, the smaller I_D_/I_G_ value of Coqpy‐pyr@CNT‐O indicates a stronger interaction between Coqpy‐pyr and CNT‐O. Besides, an up‐shift of the D and G bands for Coqpy@CNT‐O and Coqpy‐pyr@CNT‐O are observed, indicative of electronic transfer between the molecular complexes and CNT‐O through *π–π* interaction and possible coordination effect.^[^ ^36^ ^]^ Such a shift was also observed upon immobilizing Coqpy onto CNT‐N (Figure S7b, Supporting Information). The lack of identifiable peak shift for Coqpy@CNT reflects the relatively weak interaction between Coqpy and CNT (Figure S7a, Supporting Information), suggesting the presence of covalent bonding between the cobalt center and the carboxyl/amido groups on MWCNT. This bonding can enhance the interaction between the molecular complex and MWCNT.

The XPS survey spectra of Coqpy@CNT‐O and Coqpy‐pyr@CNT‐O indicate the co‐existence of C, O, N, and Co elements (Figure S8, Supporting Information). The Co 2p spectrum of Coqpy can be deconvoluted into two peaks at 780.7 and 796.1 eV with two shake‐up satellite peaks (Figure 1d), which can be assigned to the Co 2p_3/2_ and Co 2p_1/2_ of Co^2+^, respectively.^[^ ^37^ ^]^ The imperceptible difference in Co 2p and N 1s XPS spectra between Coqpy and Coqpy‐pyr indicates a negligible influence of the peripheral pyrene unit on the Co electronic state (Figure S12, Supporting Information). The shift to higher energy of Co 2p signal for Coqpy@CNT‐O and Coqpy‐pyr@CNT‐O versus Coqpy suggests an electronic interaction between the molecular catalysts and CNT‐O, resulting in a higher‐valence state of Co sites.^[^ ^38^ ^]^ Consistently, the peak corresponding to Co─N for Coqpy@CNT‐O and Coqpy‐pyr@CNT‐O in the deconvoluted N 1s spectra shifts from 399.7 to 400.2 eV (Figure 1e). In addition, the shift to higher energy of the signal attributed to the axial Cl ligand for Coqpy‐pyr@CNT‐O further demonstrates the difference in the electronic states of the Co centers (Figure S12c, Supporting Information). The decrease of the Cl/Co atom ratio from 2:1 to 1:1 upon binding the Co complexes to CNT‐O indicates that the carboxyl group coordinates to the Co center in a perpendicular fashion via substituting one Cl ligand (Table S1, Supporting Information). The deconvoluted O 1s spectrum indicates the coexistence of C═O (531.5 eV), C─O─C (532.3 eV), C─OH (533.1 eV), COOH (534.1 eV), and H_2_O or O_2_ adsorbed on the surface (536.3 eV)^[^ ^39^, ^40^ ^]^ (Figure S13, Supporting Information). Upon immobilizing Coqpy and Coqpy‐pyr, the peak assigned to carboxyl of CNT‐O slightly shifts to lower energy, strongly suggesting binding of the carboxyl oxygen to the Co center. Different from Coqpy@CNT‐O, there is nearly no shift of the Co 2p and N 1s peaks for Coqpy@CNT, thus the Co chemical state is nearly unaltered (Figure S14, Supporting Information). The Co 2p signals of Coqpy@CNT‐N are relatively weak and a slight shift to higher energy was observed (Figure S15, Supporting Information). Based on the above results, it can be concluded that the functional groups on MWCNT could regulate the electronic environment of molecular Co active sites by axial coordination effect.

**Figure 2:**
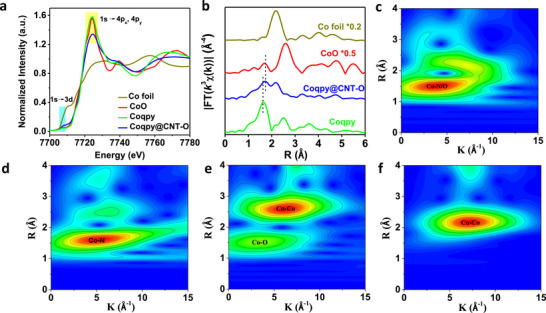
XAS of Coqpy@CNT‐O, Coqpy, CoO, and Co foil. a) Co K‐edge XANES spectra and b) FT‐EXAFS spectra. Wavelet transform EXAFS contour plots of c) Coqpy@CNT‐O, d) Coqpy, e) CoO, and f) Co foil.

To further investigate the coordination environment and electronic structure of the Co species, X‐ray absorption near edge structure (XANES) spectroscopy and extended X‐ray absorption fine structure (EXAFS) spectroscopy were performed. In the Co K‐edge XANES spectra (**Figure** **2**a), Coqpy@CNT‐O displays a similar absorption peak around 7707.7 eV as Coqpy, which originates from the dipole‐forbidden 1s to 3d transition.^[^ ^3^, ^31^ ^]^ On the other hand, the increased peak intensity of Coqpy@CNT‐O indicates a more distorted Co─N_4_ structure of immobilized Coqpy due to the axial coordination of COOH to Co. The peak around 7724 eV corresponds to the 1*s* → 4*p_x_*, *4p_y_* transition, whose peak position is associated with the valence state of cobalt.^[^ ^26^ ^]^ The slight shift of the peak position of Coqpy@CNT‐O to higher energy compared with Coqpy indicates a higher oxidation state of Co in Coqpy@CNT‐O arising from charge transfer from Coqpy to CNT‐O, in line with XPS data.

The Fourier‐transformed (FT) EXAFS spectrum of Coqpy displays one prominent peak at 1.63 Å (without phase shift correction), corresponding to the Co─N first coordination shell (Figure 2b). The FT‐EXAFS spectrum of Coqpy@CNT‐O exhibits a predominant peak at 1.73 Å, which is shifted compared with Coqpy and is close to that of CoO (1.70 Å), indicating the presence of additional Co─O scattering path other than Co─N bond. This Co─O bond should arise from the coordination of Co with O atoms of carboxyl groups on CNT─O. The peak at 2.17 Å assigned to the Co─Co bond in Coqpy@CNT─O is likely due to residual Co nanoparticles wrapped in CNT─O, which has been confirmed by TEM. The wavelet transform (WT) EXAFS analysis was further conducted to give additional information on the coordination structure of the samples (Figure 2c–f). The intensity maximum at ≈ 4 Å^−1^ for Coqpy@CNT─O resembles those of Coqpy and CoO, which can be assigned to the Co─N and Co─O coordination in the first coordination shell, indicating the presence of essentially monodispersed Co sites for the immobilized Coqpy. The signal region of Co─Co (≈ 7 Å^−1^) for Coqpy@CNT─O comes from Co nanoparticles wrapped in CNT─O. In summary, in addition to *π–π* interactions and through‐space orbital coupling, the carboxyl groups on CNT─O contribute to binding with Coqpy via axial coordination.

### 2.2 Electrocatalytic CO_2_RR

**Figure 3:**
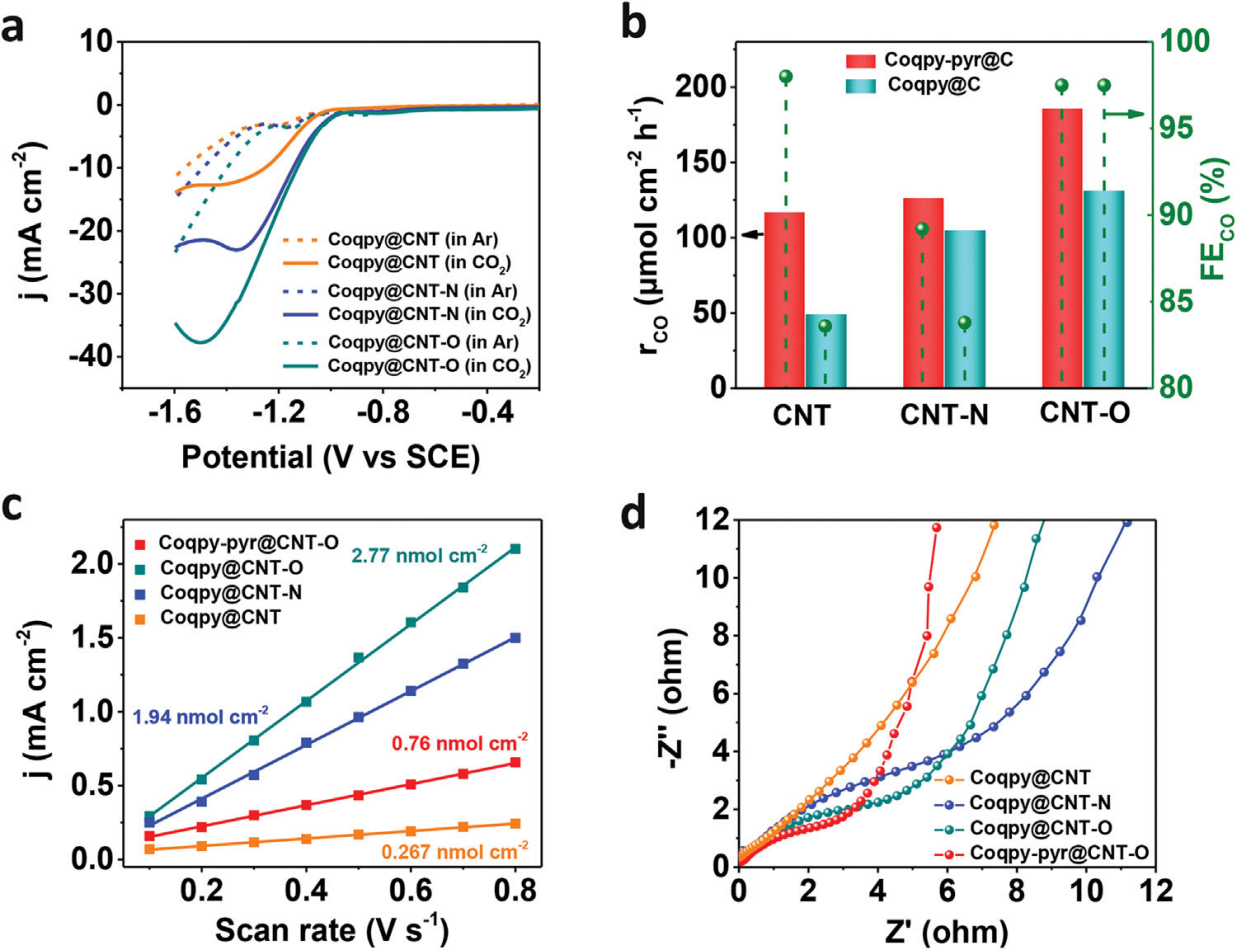
Electrocatalytic CO_2_RR performance of various MWCNT immobilized Co complexes. a) LSV of the hybrid catalysts. b) CO generation rates (r_CO_) and Faradaic efficiency (FE_CO_) for different hybrid catalysts at −1.2 V versus SCE. c) Variation of the peak current densities of Co^II^/Co^I^ redox wave versus scan rates. d) Nyquist plots for the hybrid catalysts. Conditions: 0.5 m KHCO_3_, SCE reference electrode, pH 7.2 (CO_2_), 8.8 (Ar), 100 mV s^−1^ scan rate for LSV.

The electrochemical properties of the samples were investigated using a customized single electrolytic cell. The CV of Coqpy@CNT coated on carbon paper in 0.5 m KHCO_3_ aqueous solution under argon (pH 8.8) features a Co^II^/Co^I^ couple (at ≈ −0.4 V vs SCE) and an qpy‐centered reduction wave (at −0.9 V vs SCE) (Figure S16, Supporting Information), similar to previous report.^[^ ^32^ ^]^ A catalytic wave occurs at more negative potential due to hydrogen evolution reaction (HER). Similar results were observed for Coqpy/Coqpy‐pyr@CNT‐O/CNT‐N (Figures S16 and S17, Supporting Information). Linear correlations were found between the peak current densities of [Co^I^qpy]/[Co^I^qpy^•−^] couple with scan rates, which is the characteristic of electrode‐confined complexes (Figures S18–S25, Supporting Information).^[^ ^41^ ^]^ The CV of Coqpy@CNT─O under CO_2_ shows that the catalytic current arises after the generation of two‐electron reduced active species Co^I^qpy^•−^. As revealed by linear sweep voltammetry (LSV) (**Figure** **3**a), compared with Coqpy@CNT and Coqpy@CNT─N, Coqpy@CNT─O exhibits a higher current density, indicating better catalytic activity for CO_2_RR. The same trend is also observed for the immobilized Coqpy‐pyr samples (Figure S26, Supporting Information), manifesting the role of the carboxyl group on CNT─O for promoting electrocatalytic CO_2_RR. The interaction of Co ions with the functional groups on MWCNT was measured with CoCl_2_/KCl solution to exclude the π interaction effect of ligands. As shown in Figure S27 (Supporting Information), the redox response of Co on CNT─O is higher than CNT and CNT─N, indicating the binding of carboxyl to Co. This bonding effect is beneficial for electron communication and potentially increases the number of electrochemically active immobilized Co complex molecules.

Controlled‐potential electrolysis (CPE) was performed at −1.2 V vs SCE for the hybrid catalysts. The CO generation rates (r_CO_) by Coqpy@CNT─O and Coqpy@CNT─N are respectively ≈ 2.6 and twofold higher than that of Coqpy@CNT (Figure 3b). Notably, Coqpy@CNT─O has a markedly higher FE_CO_ versus Coqpy@CNT─N (97.5% vs 83.8%). The same trend is observed for Coqpy‐pyr, with Coqpy‐pyr@CNT─O achieving the highest r_CO_ of 186.2 µmol cm^−2^ h^−1^ (r_CO_ of 117.3 and 126.8 µmol cm^−2^ h^−1^ for Coqpy‐pyr@CNT and Coqpy‐pyr@CNT─N, respectively). Moreover, the catalytic performances of Coqpy‐pyr immobilized on all the tested MWCNT are superior than the Coqpy hybrid catalysts. The TON_CO_ for Coqpy@CNT─O is also higher than those of Coqpy@CNT and Coqpy@CNT─N (Figure S29, Supporting Information), further indicating that the Coqpy coordinated to the surface carboxyl groups are more efficient toward electrocatalytic CO_2_RR.

To understand the role of the functional groups in facilitating CO_2_RR, their impact on the loading of molecular catalysts were studied. Despite the same molecular catalyst concentration in the initial ink, the actual loadings on the electrode for the various MWCNTs are different due their different interactions with the electrode. The electroactive amounts of the molecular catalysts obtained from the variation of Co^II^/Co^I^ peak current versus scan rates are 0.27, 1.94, and 2.77 nmol cm^−2^ for Coqpy@CNT, Coqpy@CNT─N, and Coqpy@CNT─O, respectively (Figure 3c; Figure S30, Supporting Information), which accounts for 1 to 16% of the total loading determined by ICP‐MS (Table S2, Supporting Information). The large deviation between the actual and electroactive Co amount for Coqpy@CNT and Coqpy@CNT─N is probably due to the relatively weak interaction of Coqpy with CNT/CNT‐N and the formation of inactive catalyst agglomerates, resulting in limited electron transport and active sites accessibility. The charge transfer resistance revealed by electrochemical impedance spectroscopy follows the order: Coqpy@CNT‐O < Coqpy@CNT‐N < Coqpy@CNT (Figure 3d), which correlates with their catalytic performances. Therefore, the better catalytic conversion of CO_2_ for Coqpy@CNT‐O is attributed to the higher number of electroactive Co sites and favorable electron transfer through axial Co─O bonding.

**Figure 4:**
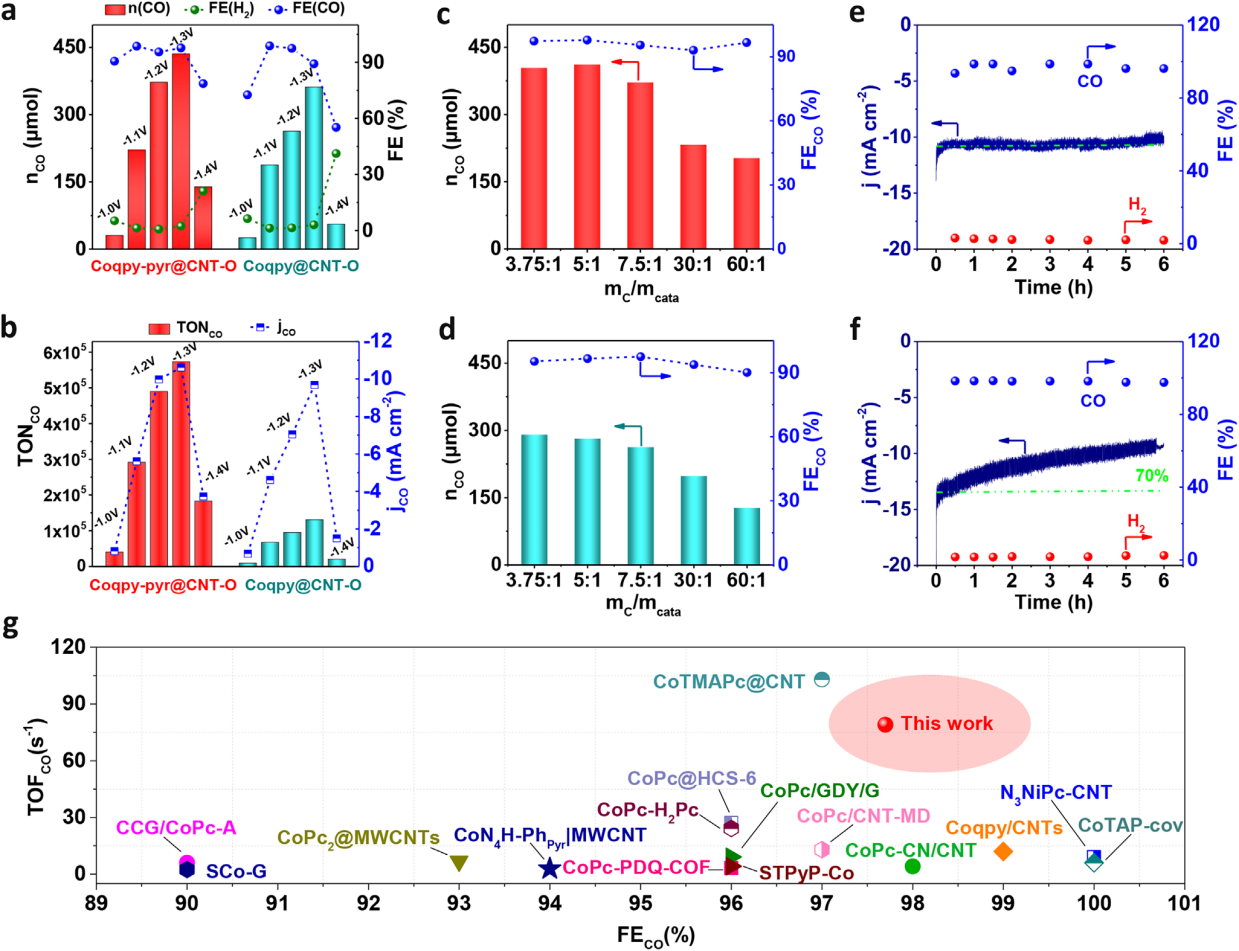
Electrocatalytic CO_2_RR performance comparison of Coqpy‐pyr@CNT─O, Coqpy@CNT─O, and reference catalysts. a) CO yield (n_CO_) and FE, b) CO patrial current densities (j_CO_) and turnover number (TON_CO_ based on the electroactive Co sites) catalyzed by Coqpy‐pyr@CNT─O and Coqpy@CNT─O at different potentials. n_CO_ and FE_CO_ of (c) Coqpy‐pyr@CNT─O and d) Coqpy@CNT─O at different catalyst mass ratios. Long‐term stability tests of CO_2_RR over e) Coqpy‐pyr@CNT─O and f) Coqpy@CNT─O. g) Performance comparison of Coqpy‐pyr@CNT─O with previously reported immobilized molecular catalysts for CO_2_RR. Conditions: 0.5 m KHCO_3_, SCE reference electrode, pH 7.2, −1.2 V versus SCE for c‐f.

The effect of the pyrene group of Coqpy‐pyr on CO_2_RR was studied under various conditions (different applied potentials and catalyst mass ratios). The current density recorded on CV of Coqpy‐pyr@CNT‐O is higher than that of Coqpy@CNT‐O (Figure S32, Supporting Information), even with lower Coqpy‐pyr loading, indicative of the higher activity of Coqpy‐pyr@CNT‐O for CO_2_RR. Coqpy‐pyr@CNT‐O delivers a higher n_CO_ and CO partial current density (j_CO_ = j_total_ × FE_CO_) at all applied potentials (**Figure** **4**a,b). The FE_CO_ of Coqpy‐pyr@CNT‐O remains above 90% from −1.0 to −1.3 V versus SCE, and only decreases to ≈ 80% at more negative potentials. On the other hand, Coqpy@CNT‐O exhibits reduced FE_CO_ of 72.6% and 55.2% at −1.0 and −1.4 V versus SCE. In addition, the catalytic performances of Coqpy‐pyr@CNT/CNT‐N are also superior than the corresponding Coqpy hybrid catalysts (Figure 3b). For Coqpy‐pyr@CNT‐O, the lower loading as compared to that of Coqpy@CNT‐O (Table S2, Supporting Information) is mainly due to the nonplanar structure and large molecular dimension of Coqpy‐pyr. Coqpy‐pyr@CNT‐O exhibits a maximum TON_CO_ of 5.7 × 10^5^ (based on the electroactive Co sites) at −1.3 V versus SCE during 2 h CPE (Figure 4b), corresponding to a turnover frequency (TOF_CO_) of 79 s^−1^, which is ∼ 4.4‐fold higher than those of Coqpy@CNT‐O (TON_CO_ and TOF_CO_ of 1.3 × 10^5^ and 18 s^−1^ respectively). The TON of Coqpy‐pyr@CNT‐O according to the actual Co loading (assuming all the molecules are active) is also higher than that of Coqpy@CNT‐O (5.3 × 10^4^ vs 2.1 × 10^4^) (Figure S33, Supporting Information). Moreover, Coqpy‐pyr@CNT‐O outperforms Coqpy@CNT‐O for CO_2_RR under various Co complex mass ratios (Figure 4c,d). The slower growth of CO production at high loading primarily stems from the molecular saturation adsorption on CNT─O. The absence of decreased conductivity for Coqpy‐pyr@CNT‐O and Coqpy@CNT─O at higher catalyst loadings^[^ ^35^ ^]^ can be supported by the similar redox peak separation as a function of scan rates, which relates to the electron transfer rate^[^ ^18^ ^]^ (Figures S23 and S25, Supporting Information). The slightly lower charge transfer resistance for Coqpy‐pyr@CNT‐O than that of Coqpy@CNT‐O also emphasizes the efficient electron transmission in Coqpy‐pyr@CNT─O (Figure 3d). To the best of our knowledge, the high TOF reaching 79 s^−1^ (η = 523 mV) given by Coqpy‐pyr@CNT‐O is among the most active metal complexes‐based CO_2_RR catalysts (Figure 4g; Table S3, Supporting Information). Also, Coqpy‐pyr@CNT─O maintains a high CO FE of ≈92% even at current densities of 100 and 150 mA cm^−^^2^ (Figure S34, Supporting Information).

The durability of the catalysts was evaluated at −1.2 V versus SCE for 6 h CPE (Figure 4e,f). The total current density was nearly unchanged and FE_CO_ remained in the range of 90–100% for Coqpy‐pry@CNT‐O during the period, producing TON_CO_ of 1.6 × 10^5^ based on actual Co loading. In contrast, the current density of Coqpy@CNT‐O decreased by 30% under the same conditions. CV scans performed on Coqpy@pyr‐CNT─O after CPE showed almost identical redox behavior with the fresh one (Figure S35, Supporting Information), highlighting that Coqpy‐pyr molecules are well‐retained within the film. On the contrary, the markedly diminished redox waves in the range of −0.7–−1.0 V versus SCE for Coqpy@CNT─O suggests the stripping of Coqpy from the electrode surface. XPS characterization of the electrode after the 6 h CPE exhibits a significantly larger decrease in Co 2p signal intensity for Coqpy@CNT─O than that of Coqpy‐pyr@CNT─O (Figure S36, Supporting Information). Compared with Coqpy‐pry@CNT─O, Coqpy@CNT─O undergoes more catalyst leaching (65.8% vs 9.7% of Co from XPS analysis) (Table S4, Supporting Information). The maintenance of the Co oxidation state and chemical environment of Coqpy‐pry@CNT‐O after electrolysis is also supported by the absence of any new peak. The uniformly dispersed Co sites in TEM images of Coqpy‐pry@CNT‐O after electrolysis also indicates the presence of coordinated Co centers (Figure S37, Supporting Information). The high stability of Coqpy‐pry@CNT‐O was also corroborated by the electrolysis and post‐catalysis characterization at high current densities (Figures S38–S40, Supporting Information). The higher electrocatalytic CO_2_RR efficiency and stability of Coqpy‐pyr@CNT‐O than Coqpy@CNT‐O can be attributed to the faster interfacial electron transfer and stronger binding between Coqpy‐pyr and CNT‐O via reinforced *π–π* interaction brought by the pyrene moiety. Moreover, a Tafel slope of 117 mV dec^−1^ was determined for Coqpy‐pyr@CNT‐O (Figure S41, Supporting Information), indicating the initial one‐electron transfer or proton‐coupled electron transfer (PCET) process is the rate‐determining step (118 mV dec^−1^).^[^ ^23^, ^42^, ^43^ ^]^

Control experiments using only MWCNT under CO_2_ operated at −1.2 V versus SCE showed very low current density of ≈ −0.5 mA cm^−2^ (Figure S44, Supporting Information), with H_2_ being the main product (14.8, 10.8, and 9.9 µmol for CNT, CNT‐N, and CNT‐O, respectively) while no CO was detected, indicating Coqpy is the active center for CO_2_RR. Minor amounts of CO were detected for Coqpy@CNT‐O (22.1 µmol) and Coqpy‐pyr@CNT‐O (34.2 µmol) under Ar, which should come from the supporting electrolyte KHCO_3_, it accounts for ≈10% of CO produced in CO_2_‐saturated solution. And no CO was detected under Ar in 0.5 m KCl. In isotope labeling experiments, only ^13^CO was detected when using ^13^CO_2_ as the reactant in 0.5 m KCl, verifying CO being from the CO_2_ reduction (Figure S45, Supporting Information). In addition, Coqpy and Coqpy‐pyr supported on MWCNT showed much higher catalytic activity for CO_2_RR compared with the unsupported complexes: CO production is 38 and tenfold higher upon immobilization of Coqpy and Coqpy‐pyr on CNT‐O, respectively. Higher FE_CO_ are also achieved upon immobilization of Coqpy (32.2% vs 97.5%) and Coqpy‐pyr (74.7% vs 97.5%). Notably, the homogeneous Coqpy and Coqpy‐pyr complexes display negligible electrocatalytic CO_2_RR activity with no CO detected at −1.3 V versus SCE (Figure S46, Supporting Information). The higher catalytic efficiencies of MWCNT‐supported molecular complexes are attributed to the strong electronic interaction and improved accessibility of active sites benefitted from the molecularly dispersed Co complexes.

### 2.3 Reaction Mechanism

**Figure 5:**
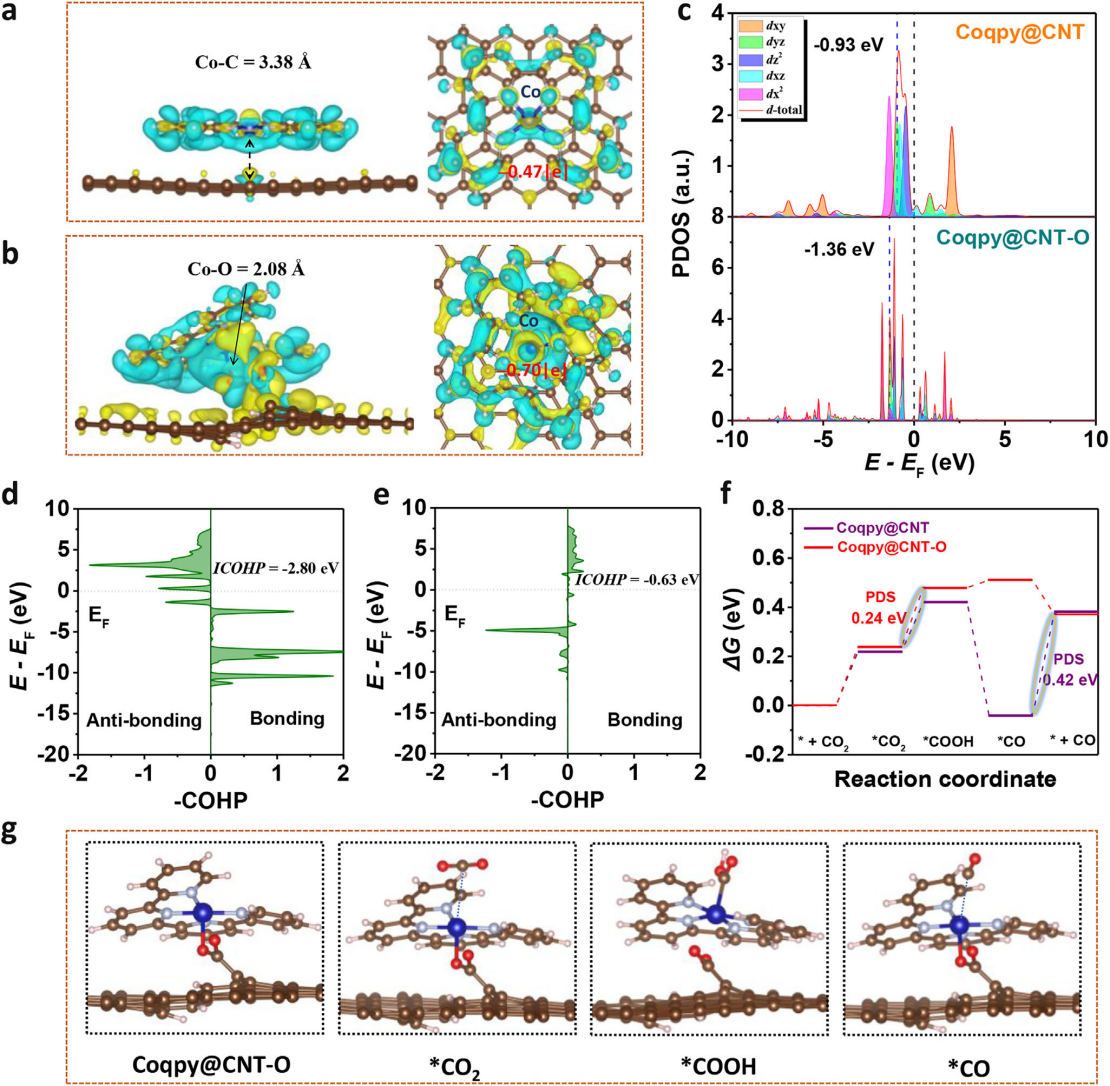
Structure analysis and mechanistic study by DFT calculations. Top and side views of molecular configuration and charge density differences of Coqpy immobilized on (a) CNT and (b) CNT─O (Yellow and cyan represent electron accumulation and depletion, respectively). c) Co 3d PDOS for Coqpy@CNT and Coqpy@CNT─O. Crystal orbital Hamilton population (COHP) of CO adsorbed on (d) Coqpy@CNT and (e) Coqpy@CNT─O. f) Gibbs free energy diagrams for CO_2_RR over Coqpy@CNT─O and Coqpy@CNT (pH 7, U = −0.53 V vs RHE). g) Optimized geometry of intermediates during CO_2_RR over Coqpy@CNT─O (C: brown; N: pale blue; O: red; H: white; Co: navy).

DFT calculations have been performed to provide more insights into electrocatalytic properties of Coqpy immobilized on carboxyl‐modified MWCNT. The carboxyl oxygen on CNT─O was found to be effective binding sites to immobilize Coqpy via axial Co─O coordination, which is evidenced by its shorter Co─O bond length and the rather extensive charge redistribution between the Co center and the carboxyl group (**Figure** **5**a,b). As a result, Co has a higher oxidation state in Coqpy@CNT─O compared to Coqpy@CNT, as indicated by the more extensive electron transferred from Co to CNT─O (0.70 |e|) than to CNT (0.47 |e|) from the Bader charge analysis. This is also consistent with the XPS and XANES results. These results show that the carboxyl group affords a strong binding site for Co, resulting in stable immobilization and more extensive electron delocalization via Co─O bonds. The partial density of states (PDOS) of Co 3d (Figure 5c) shows that the Co─O coordination for Coqpy@CNT─O results in a downshift of the d‐band center of Co atoms. Such electronic modification could tune the binding and activation of reaction intermediates.^[^ ^44^, ^45^, ^46^ ^]^ Thus, a relatively weak binding interaction of *CO, which facilitates CO release from the Co site of Coqpy@CNT─O is obtained, consistent with the free energy analysis (Figure 5f). Crystal orbital Hamilton population (COHP) analysis was conducted to quantify the bonding strength of Co for *CO species (Figure 5d,e). The smaller integrated COHP (ICOHP) absolute value of the Co─C bond for *CO adsorbed on Coqpy@CNT─O further reveals a weaker adsorption. As such, the potential determining step (PDS) for Coqpy@CNT‐O is different from that of Coqpy@CNT, with the highly endergonic PCET to generate *COOH process being the PDS (Figure 5f). This PDS energy barrier (0.24 eV) is lower than that of Coqpy@CNT (0.42 eV, *CO → CO), illustrating the impact of axial O coordination for CO_2_RR.

Combining the characterization, electrocatalytic, and DFT results, the high catalytic efficiency of CNT─O immobilized Co complex can be ascribed to the following: i) the efficient coordination of carboxyl to Co enables more electroactive Co complex molecules to be loaded on the electrode surface; ii) the Co─O bond can serve as a bridge to facilitate electron transfer from electrode to Co complex; iii) the resultant higher‐valence state of Co can accelerate the reaction kinetics and lowers the energy barrier for CO_2_RR.^[^ ^47^ ^]^

## 3 Conclusion

In summary, upon using coordination and enhanced *π–π* interaction effects, we have significantly enhanced the efficiency and durability of cobalt quaterpyridine@MWCNT hybrids for electrocatalytic CO_2_ reduction. We have appended a highly aromatic pyrene moiety to the qpy ligand, which resulted in much stronger *π–π* interactions of the cobalt complex with MWCNT. In addition, we have functionalized MWCNT with carboxyl or amide groups. These functional groups, particularly the carboxyl groups, provide additional binding of the Co complex to the MWCNT surface through the formation of the Co─O bond. Both enforced *π–π* interactions and Co─O bonding enable more electroactive Co complex attached on MWCNT, together with strong electronic coupling between Co and MWCNT. The modified Co electronic configuration enhances the reaction kinetics and lowers the energy barrier for CO desorption. As a result, Coqpy‐pyr@CNT─O is found to be among the best catalysts based on molecular complexes, with TOF of 79 s^−1^, TON of 5.7  × 10^5^, FE close to 100%, and the hybrid catalyst is highly stable. This dual immobilization strategy is a significant step toward a practical system for the electrocatalytic reduction of CO_2_ in aqueous solutions.

## Conflict of Interest

The authors declare no conflict of interest.

## Supporting information



Supporting Information

## Data Availability

The data that support the findings of this study are available from the corresponding author upon reasonable request.
