# Supplementary material for: Cobalt Pyrene‐Quaterpyridine Molecular Complex Immobilized on Functionalized Multi‐Walled Carbon Nanotubes as a Robust Hybrid Catalyst for Efficient Electrochemical Reduction of CO2
Source: Adv Sci (Weinh). 2025 Sep 16;12(44):e09854. doi: 10.1002/advs.202509854 (PMC12667495; doi:10.1002/advs.202509854)
Supplement: Supplementary file 1 — Supporting Information [file ADVS-12-e09854-s001.docx]

Cobalt Pyrene-Quaterpyridine Molecular Complex Immobilized on Functionalized Multi-Walled Carbon Nanotubes as a Robust Hybrid Catalyst for Efficient Electrochemical Reduction of CO_2_

Yue Wei,^[a]^ Wangxiyue Yi,^[a]^ Lingjing Chen,^[a]^ Huatian Shi,^[a]^ Marc Robert,*^[b]^ Tai-Chu Lau,*^[a,^ ^c]^ Gui Chen*^[a]^

[a] School of Environment and Civil Engineering, Research Center for Eco-environmental Engineering, Guangdong Provincial Key Laboratory of Intelligent Disaster Prevention and Emergency Technologies for Urban Lifeline Engineering, Dongguan University of Technology, Dongguan, Guangdong 523808, P. R. China

[b] Sorbonne Université, CNRS, Institut Parisien de Chimie Moléculaire, Paris F-75005, France; Institut Universitaire de France (IUF), Paris F-75005, France

[c] Department of Chemistry, City University of Hong Kong, Tat Chee Avenue, Kowloon Tong, Hong Kong 999077, P. R. China

*Corresponding authors: marc.robert@sorbonne-universite.fr; [bhtclau@cityu.edu.hk](mailto:bhtclau@cityu.edu.hk); chengui@dgut.edu.cn

**Chemicals.**

Cobalt (II) chloride hexahydrate (Acros Organics, 98%), methanol (MeOH, reagent grade, Sigma-Aldrich), isopropanol (reagent grade, Sigma-Aldrich), ethanol (EtOH, AR, Tianjin Damao Chemical Reagent Co., Ltd.), glycol (AR, Tianjin Damao Chemical Reagent Co., Ltd.), 1-pyrene butyric acid (Shanghai 9 Ding Chemistry Co., Ltd., 98%), benzotriazole-1-yl-oxytripyrrolidinophosphonium hexafluorophosphate (PyBOP, J&K, 99%) and ethyldiisopropylamine (DIEA, J&K, 99%), acetonitrile (99.9%, Energy-Chemical), N, N-dimethylformamide (DMF, 99.9%, Energy-Chemical), Nafion solution (5 wt% in a mixture of lower aliphatic alcohols and water, Sigma-Aldrich) were used without further purification. All MWCNTs were purchased from Nanjing XFNANO Material Tech Co.. Carbon fiber paper (CFP, HCP030N) was provided by Shanghai Hesen Electric Co., Ltd. 4-bromo-2,2′:6′,2″:6″,2‴-quaterpyridine and Co quaterpyridine (Coqpy) were synthesized according to literature.^[^[^1^](#_ENREF_1)^]^

**Synthesis of Coqpy-pyr.**

**Qpy-Ph-NH_2_ preparation.**

4-([2,2':6',2'':6'',2'''-quaterpyridin]-4-yl)aniline hydrochloride (qpy-Ph-NH_2_·HCl) was synthesized according to the literature method.^[^[^2^](#_ENREF_2)^]^

**Qpy-pyr (L) preparation.**

1-pyrene butyric acid (35 mg, 0.12 mmol), benzotriazole-1-yl-oxytripyrrolidinophosphonium hexafluorophosphate (PyBOP, 104 mg, 0.02 mmol) and ethyldiisopropylamine (DIEA, 52 mg, 0.4 mmol) were added to 5 mL dried DMF and the mixture was stirred for 12 h under argon. Then, 4-([2,2':6',2'':6'',2'''-quaterpyridin]-4-yl)aniline hydrochloride (qpy-Ph-NH_2_·HCl) (43.7 mg, 0.1 mmol) was added and reacted for 72 h. After removing DMF, the residues were washed with water and isopropanol. Finally, the crude product was dried to obtain the N-(4-([2,2':6',2'':6'',2'''-quaterpyridin]-4-yl)phenyl)-4-(pyren-1-yl)butanamide (qpy-pyr, L) ligand and was used to prepare the cobalt complex without further purification. Yield: 60 mg (89%). ^1^H NMR (400 MHz, DMSO-d6): δ 10.24 (s, 1H), 8.93 (s, 1H), 8.85-8.79 (m, 3H), 8.77 (d, J = 8.0 Hz, 1H), 8.70 (d, J = 8.0 Hz, 1H), 8.62 (d, J = 8.0 Hz, 1H), 8.52 (d, J = 8.0 Hz, 1H), 8.45 (d, J = 8.0 Hz, 1H), 8.31 - 8.221 (m, 5H), 8.15 (d, J = 4.0 Hz, 2H), 8.08 (d, J = 8.0 Hz, 2H), 8.05 - 7.99 (m, 3H), 7.86 (d, J = 4.0 Hz, 2H), 7.57 (t, J = 8.0 Hz, 1H), 3.43 (d, J = 8.0 Hz, 2H), 2.55 (d, J = 8.0 Hz, 2H) , 2.16 (d, J = 8.0 Hz, 2H).

**Coqpy-pyr preparation.** A mixture of ligand qpy-pyr (33 mg, 0.05 mmol) and CoCl_2_·6H_2_O (24 mg, 0.1 mmol) in 20 mL MeOH was sonicated for 2 h and followed by stirring at room temperature for 48 h. After evaporating the organic solvent, the residue was washed with water, isopropanol, and dichloromethane to give Coqpy-pyr (CoLCl_2_) as a yellow solid. Yield: 30 mg (37%). TOF-MS in MeOH containing 0.1% HCOOH as the mobile phase: m/z 775.1974, [CoL^2+^ + HCO_2_^-^]^+^ (Figure S1). CoLCl_2_∙1.5H_2_O Anal. Calcd. (Found) for C_46_H_36_Cl_2_CoN_5_O_2.5_: C, 66.67 (66.69), H, 4.38 (4.16), N, 8.45 (8.49).

**Scheme S1.** Synthesis of Coqpy-pyr.

**Characterization.** Electrospray ionization mass spectrometry (ESI-MS) was performed on a PerkinElmer NexION 300X instrument. X-ray photoelectron spectroscopy (XPS) spectra were recorded on a Thermo Fisher ESCALAB Xi+ XPS spectrometer and were calibrated using a C 1s signal of 284.8 eV. The Co ion loading on the electrode was obtained using inductively coupled plasma mass spectrometry (ICP-MS, Agilent 7800). The high-resolution transmission electron microscopy (HR-TEM) images were acquired on a JEOL JEM-2010 instrument equipped with an energy dispersive spectrometer (EDS) detector. The UV-vis diffuse reflectance spectroscopy (UV-vis DRS) measurement was conducted on a Perkin-Elmer lambda 750s spectrometer. Raman tests of samples were performed on a Laser confocal Raman instrument (HORIBA XploRA PLUS) with an excitation wavelength of 532 nm. The X-ray absorption spectroscopy (XAS) measurements including X-ray absorption near edge structure (XANES) and extended X-ray absorption fine structure (EXAFS) (Co K-edge) were carried out at 5S1 X-ray absorption beamline of Aichi Synchrotron Radiation Center. This beamline adopted a double-bounce channel-cut Si (111) monochromator for mono-beam XAS. The spectra of the samples were recorded in fluorescence mode while those of references (Co foil, CoO) were collected in transmission mode. The photon flux on the sample ranges from 2.3×10^10^ ~ 5×10^9^ photon/sec for X-ray energy from 17 keV ~ 22 keV in high energy mode. The obtained XAFS data was processed in Athena (version 0.9.26) for background, pre-edge line and post-edge line calibrations.^[^[^3^](#_ENREF_3)^]^ The Fourier transformed fitting was carried out in Artemis (version 0.9.26). For Wavelet Transform analysis, the χ(k) exported from Athena was imported into the Hama Fortran code.^[^[^4^](#_ENREF_4)^,^ [^5^](#_ENREF_5)^]^ The parameters were listed as follows: R range, 1 - 4 Å, k range, 0 - 15 Å^-1^ for samples; k weight, 3; Morlet function with κ = 10, σ = 1 was utilized as the mother wavelet to provide the overall distribution.

**Electrode preparation.** 1.5 mg of pure multiwalled carbon nanotube (MWCNT), MWCNT functionalized with carboxyl or amino (respectively abbreviated as CNT, CNT-O, and CNT-N) was dispersed in an equal volume of ethylene glycol/ethanol mixture followed by 30 min of sonication. Various volumes of Co complex (Coqpy or Coqpy-pyr) dispersion in 1:1 (v/v) ethylene glycol/ethanol (1 mg/mL) were added to the MWCNT suspension with a fixed total volume of 1.5 mL. Then the mixture was sonicated for 30 min to obtain a suspension with different MWCNT/catalyst mass ratios. Finally, 20 μL Nafion solution was added, followed by 30 min sonication to obtain the final catalyst ink. 200 μL of the ink was dropped on the CFP (1.0×1.0 cm^2^) (50 μL for each time) and dried in an infrared-ray oven for 5 min. For comparison, CFP directly immobilized Co complexes were also prepared following the same procedure only excluding the MWCNT addition.

**Electrochemical measurements.** Electrochemical CO_2_ reduction reaction (CO_2_RR) was carried out in a gas-tight customized single-compartment cell using 0.5 M KHCO_3_ (pH 7.2) aqueous solution as electrolyte. The cell was purged with CO_2_ (99.999%) for at least 50 minutes before electrolysis. For long-term electrolysis tests, high-purity CO_2_ was continuously purged into the cell with the flow rate kept constant at 20 mL/min using a mass flow controller. The cell was equipped with a saturated calomel electrode (SCE) and a platinum wire as the reference electrode and the counter electrode, respectively. All electrode potentials were reported with respect to the SCE and not corrected by iR compensation. The measured potentials can be converted to the RHE by: E (*vs.* RHE) = E (*vs.* SCE) + 0.242 V + 0.059 V × pH. The electrochemical response was recorded on a Solartron Analytical EnergyLab XM electrochemical workstation. The gas products were analyzed by GC (Agilent HP 7820A) with a thermal conductivity detector while the liquid-phase products were characterized by ionic chromatography (Dionex). The Faradaic efficiencies (FE) of CO and H_2_ were calculated using the following equation:

$$FE=\frac{2nF}{Q}$$

Where 2 is the electron number required to produce CO and H_2_, n is the molar number of products, F is the Faradaic constant (96485 C mol^-1^), and Q is the total amount of charge (C) passed through the sample during CO_2_RR.

The turnover number (TON) and turnover efficiency (TOF) for CO production were calculated as follows:

$$TON=\frac{n_{CO}}{n_{Co}}$$

$$TOF=\frac{TON}{t}$$

Where n_co_ is the generated CO mole number and n_Co_ is the total loading of Co complex determined by ICP analysis or electrocatalytic active Co amount calculated from CV scans.

For the controlled potential electrolysis (CPE) experiments conducted with continuous CO_2_ flow, the FE was calculated as below:

$${FE}_{x}=\frac{j_{x}}{j_{total}}=\frac{2\times v_{x}\times F\times100\%}{j_{total}}$$

Where j_total_ is the recorded total current density, j_x_ is the partial current density for x production, 2 is the electron number required to produce CO and H_2_, v_x_ is the molar generation rate of x (v_x_ = p×F_x_×ν/RT, where F_x_ is the volume concentration of x in the outlet gas determined by GC, p is the pressure, ν is the gas flow rate, R is the ideal gas constant of 8.314 J mol^-1^ K^-1^, and T is the temperature), F is the Faradaic constant (96485 C mol^-1^).

Turnover number (TON_CO_) was calculated as follows:

$${TON}_{CO}=\frac{Q\times{FE}_{CO(average)}}{2\times F\times n_{Co}}$$

Where Q and FE_CO_ (average) are the cumulative charge amount and the average FE_CO_ during electrocatalysis. n_Co_ is the total loading of Co complex determined by ICP analysis or electrocatalytic active Co site amount calculated from CV scans.

For the flow cell electrochemical tests, gas diffusion electrode (GDE, SGL Sigracet 39BC) coated with catalysts, a leak-free Ag/AgCl and platinum foil were used as the working, reference, and counter electrode, respectively. The working electrodes were prepared by drop-casting 200 μL of catalyst ink onto the microporous side of the GDE. The exposed electrode area was 0.5 × 2.0 cm^2^. The catholyte chamber was separated from the anolyte chamber by a Nafion 117 cation exchange membrane. Before the electrochemical test, the electrolytes (0.5 M KHCO_3_) were circulated in both the catholyte and anolyte chambers using peristaltic pumps at flow rates of 15 mL min^-1^ and 20 mL min^-1^, respectively. High-purity CO_2_ (99.999%) was purged to the gas chamber at a flow rate of 26  mL min^-1^ with a mass flow controller.

The Linear sweep voltammetry (LSV) curves were tested at a potential range from 0 to −1.6 V *vs*. SCE with a scan rate of 100 mV s^-1^. The differential pulse voltammetry (DPV) tests were conducted at an amplitude of 50 mV, pulse width of 0.05 s, pulse period of 0.5 s and sampling width of 0.0167 s. The electrochemical impedance spectroscopy (EIS) tests were performed under the frequency range from 0.01 to 100 kHz. The amount of electrochemical active Co complex (Γ, nmol cm^-2^) was calculated by plotting peak current (i_p_) corresponding to Co^I^/Co^II^ redox couple (anodic peak) under Ar atmosphere, against scan rate (ν) based on the CV curves at various scan rates. The calculation equation is shown as follows:

$$i_{p}=\frac{n^{2}\times F^{2}\times\nu\times A\times\Gamma}{4R\times T}$$

Where n is the electron transfer number for the redox wave, F is the Faradaic constant (96485 C mol^-1^), A is the electrode area (1 cm^2^), and R is the ideal gas constant of 8.314 J mol^-1^ K^-1^.

Tafel curves were plotted the logarithm of CO partial current density [log |j_CO_|] obtained from CPE experiments against overpotential.

Homogeneous electrochemical tests were carried out with 0.5 mM molecular catalyst in 4 mL electrolyte, and glassy carbon (0.0706 cm^2^) was used as the working electrode.

**Computational details.** The spin-polarized DFT calculations were performed by the Vienna ab initio simulation package (VASP)^[^[^6^](#_ENREF_6)^,^ [^7^](#_ENREF_7)^]^ with the projector augmented wave (PAW) method.^[^[^8^](#_ENREF_8)^,^ [^9^](#_ENREF_9)^]^ The generalized gradient approximation (GGA) in the form of Perdew–Burke–Ernzerhof^[^[^10^](#_ENREF_10)^]^ was used to describe the exchange-correlation effect.^[^[^11^](#_ENREF_11)^]^ The van der Waals interaction was taken into account at the DFT-D3 level. Given structural models were relaxed until the Hellmann-Feynman forces smaller than 0.02 eV/Å and the change in energy smaller than 10^-5^ eV was attained. A perfect slab model was composed of a (4 × 7) graphene (001) supercell with a vacuum layer of 30 Å. A defect model was constructed by removing two C atoms in the center and saturating the defect sites with H atoms. A 2 × 2 × 1 Monkhorst–Pack k-point mesh was used and the kinetic energy cutoff was set as 450 eV. The crystal orbital Hamilton population (COHP) analysis was implemented with the LOBSTER program.^[^[^12^](#_ENREF_12)^]^

The Gibbs free energy change (ΔG) was calculated using the computational hydrogen electrode model developed by Nørskov et al.,^[^[^13^](#_ENREF_13)^]^ in which the free energy of a pair of proton and electron was calculated as G(H^+^+ e^–^) = 1/2G(H_2_).

The ΔG calculation adopts the following equation:

Δ*G* = Δ*E*_DFT_ + Δ*E*_ZPE_ − *T*Δ*S* + Δ*G*_U_ + Δ*G*_pH_

Where Δ*E*_DFT_, ΔS, and ΔE_ZPE_ are the reaction energy change, entropy variation, and zero-point energy difference, respectively; T is the temperature (298.15 K); Δ*G*_U_ = −neU is the free energy changed by bias potential U, n is the transferred electron number. ΔG_pH_ represents the pH effect, Δ*G*_pH_ = k_B_T × pH × ln10, k_B_ is the Boltzmann constant.

The elementary steps for CO_2_RR are shown as follows:

* + CO_2_ → *CO_2_ (1)

*CO_2_ + H^+^ + e^−^ → *COOH (2)

*COOH + H^+^ + e^−^ → *CO + H_2_O (3)

*CO → * + CO (4)

where the asterisk (*) denotes the active site of catalysts.


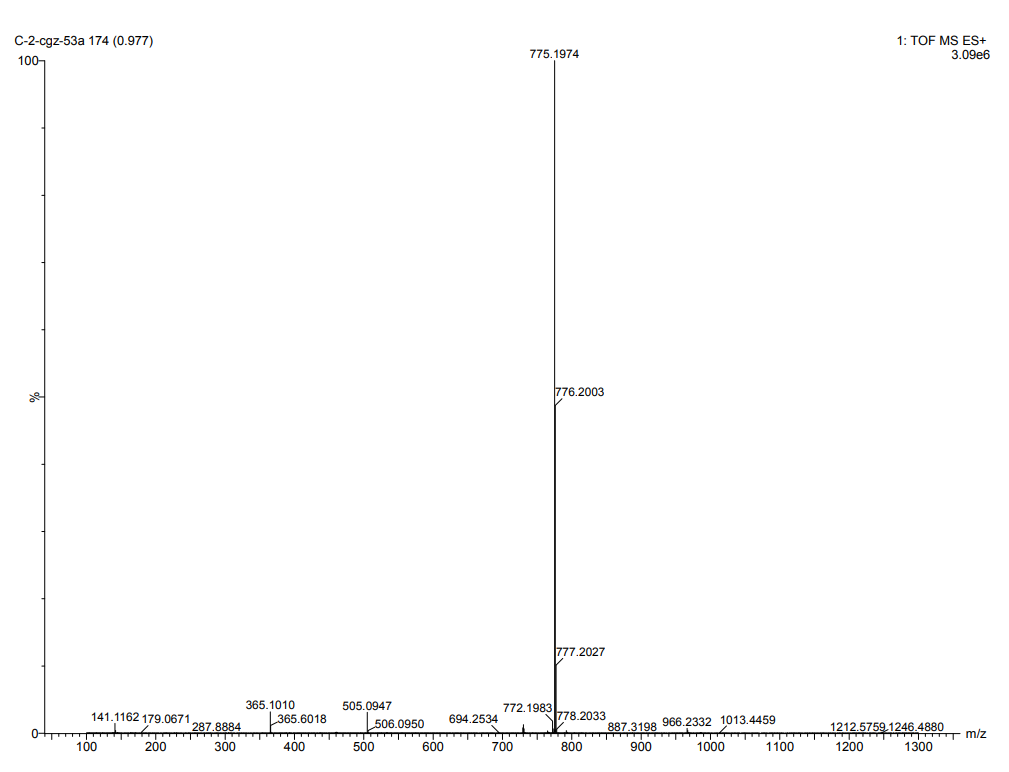


**Figure S1.** ESI-MS spectra of Coqpy-pyr in CH_3_OH containing 0.1% HCOOH as the mobile phase.

**
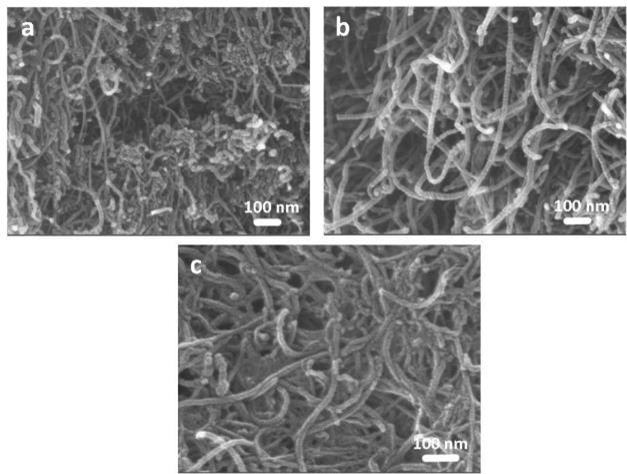
**

**Figure S2.** SEM images of Coqpy@CNT (a), Coqpy@CNT-N (b), and Coqpy@CNT-O (c).

**
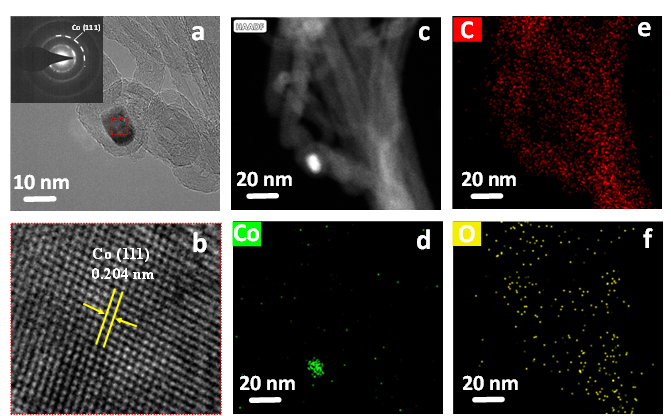
**

**Figure S3.** (a) TEM image of CNT-O exhibiting Co nanoparticles wrapped in carbon layers (the inset shows selected area electron diffraction (SAED) pattern of Co nanoparticle); (b) high-magnification TEM image of Co nanoparticle; (c-f) HAADF-STEM image and the corresponding EDS elemental mappings of CNT-O.


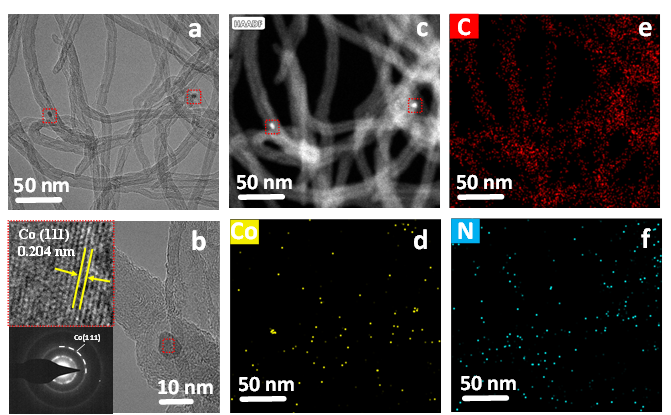


**Figure S4.** (a, b) High-magnification TEM images of Coqpy@CNT-O showcasing Co nanoparticles wrapped in carbon layers (inset of b: the [111] crystal plane (up) and SAED pattern (down) of Co nanoparticle, Co nanoparticles were labelled by red box); (c-f) HAADF-STEM image of the region containing Co particles and the corresponding EDS elemental mappings of Coqpy@CNT-O.

**
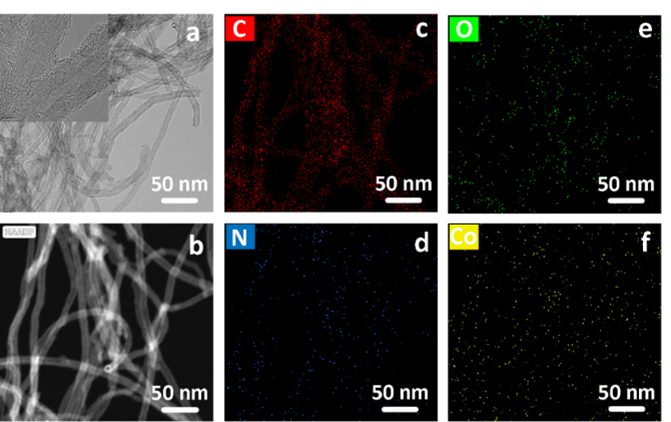
**

**Figure S5.** (a) TEM image, (b-f) HAADF-STEM image and the corresponding EDS elemental mappings of Coqpy-pyr@CNT-O.

The EDS shows the homogeneously distributed Co elements over CNT-O, proving the high dispersion of immobilized Coqpy-pyr molecules.


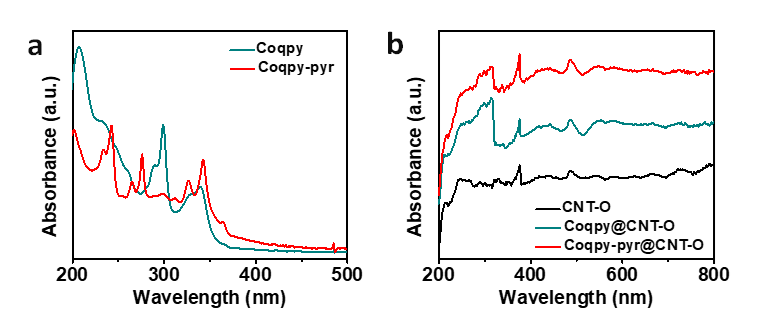


**Figure S6.** (a) UV-vis spectra of Coqpy and Coqpy-pyr dispersed in acetonitrile; (b) UV-vis DRS spectra of Coqpy@CNT-O and Coqpy-pyr@CNT-O in comparison with CNT-O.

The observed bands at 200-350 nm in the UV-vis DRS spectra of Coqpy@CNT-O and Coqpy-pyr@CNT-O, which are attributable to the characteristic absorption of Coqpy (strong absorption peaks at 207, 298, and 340 nm) and Coqpy-pyr (strong absorption bands at 242, 276, and 342 nm), indicating the successful hybridization of Co complexes and CNT-O. The variation in absorption bands for Coqpy-pyr relative to Coqpy originates from the induced pyrene moieties.

**
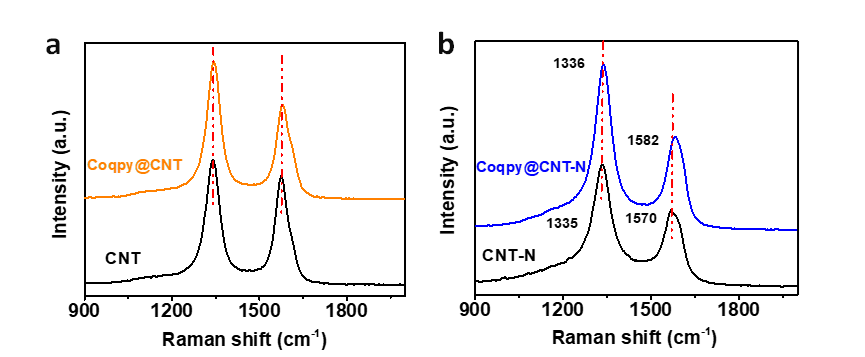
**

**Figure S7.** Raman spectra of (a) CNT and (b) CNT-N before and after immobilizing Coqpy.

**Figure S8.** XPS survey spectra of Coqpy, Coqpy@CNT-O and Coqpy-pyr@CNT-O.

The XPS survey spectra of Coqpy, Coqpy@CNT-O and Coqpy-pyr@CNT-O exhibit the co-existence of C, O, N, and Co elements while the weak signal of Co for Coqpy@CNT-O and Coqpy-pyr@CNT-O stems from their low loading in the hybrid materials. The Cl element of Coqpy stems from the counter anions.

**
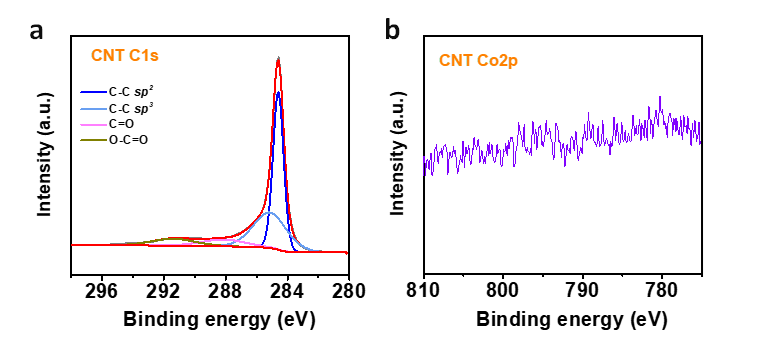
**

**Figure S9.** (a) C 1s and (b) Co 2p XPS spectra of CNT.

Nearly no Co signal is observed for pristine CNT.


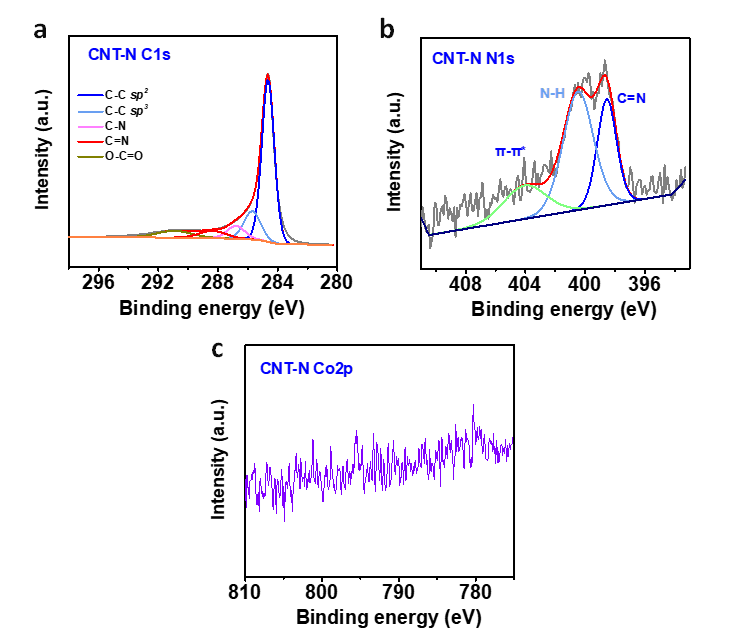


**Figure S10.** (a) C 1s, (b) N 1s, and (c) Co 2p XPS spectra of CNT-N.

The C 1s XPS spectra can be deconvoluted into five peaks, corresponding to sp^2^ carbon, sp^3^ carbon, C-N, C=N, and O-C=O, respectively. The N 1s XPS spectra can be deconvoluted into three peaks, assigning to N-H, C=N, and shake-up features, respectively. Nearly no Co signal is observed for pristine CNT-N.

**
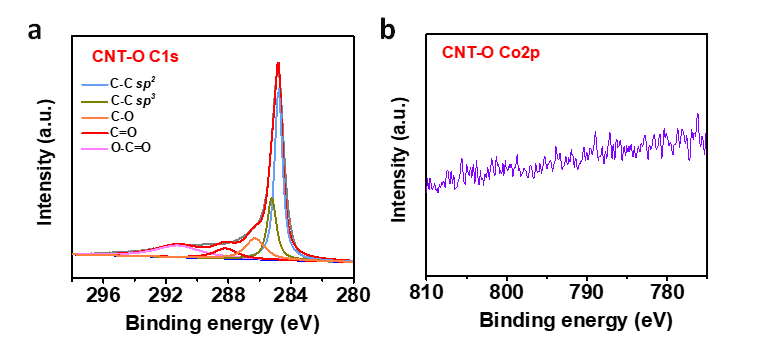
**

**Figure S11.** (a) C 1s and (b) Co 2p XPS spectra of CNT-O.

The C 1s XPS spectra can be deconvoluted into five peaks, corresponding to sp^2^ carbon, sp^3^ carbon, C-O, C=O, and O-C=O, respectively. Nearly no Co signal is observed for pristine CNT-O, which is because Co nanoparticles are encapsulated within the interior of CNT-O and XPS is superficial element-sensitive.


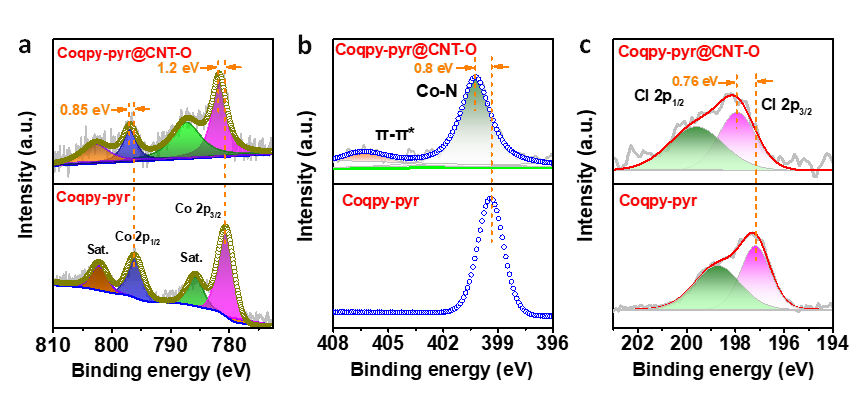


**Figure S12.** High-resolution (a) Co 2p, (b) N 1s, and (c) Cl 2p XPS spectra of Coqpy-pyr@CNT-O and Coqpy-pyr.

**Figure S13.** High-resolution O 1s XPS spectra of CNT-O, Coqpy@CNT-O and Coqpy-pyr@CNT-O.

The O 1s XPS spectra can be deconvoluted into five peaks located at 531.5, 532.3, 533.1, 534.1 and 536.3 eV, ascribable to the C=O, C-O-C, C-OH, COOH and surface-adsorbed H_2_O or O_2_, respectively. The slightly negative shift of the peak assigned to COOH of CNT-O upon immobilizing Coqpy and Coqpy-pyr implies that COOH oxygen atoms may be the primary anchoring sites for Co complexes.


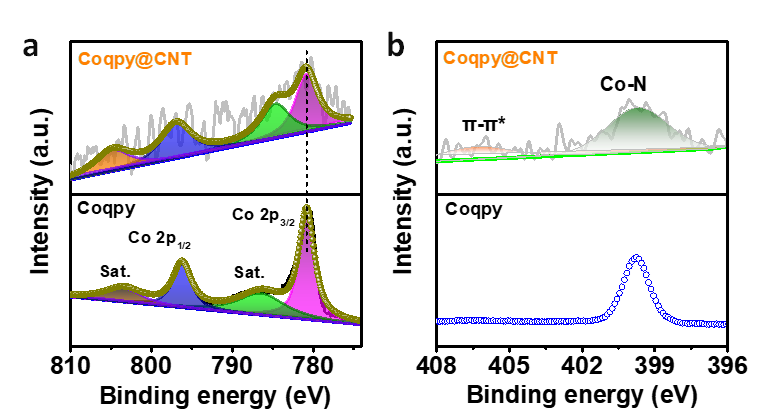


**Figure S14.** High-resolution (a) Co 2p and (b) N 1s XPS spectra of Coqpy@CNT and Coqpy.

**Figure S15.** High-resolution Co 2p XPS spectra of Coqpy@CNT-N and Coqpy.

**
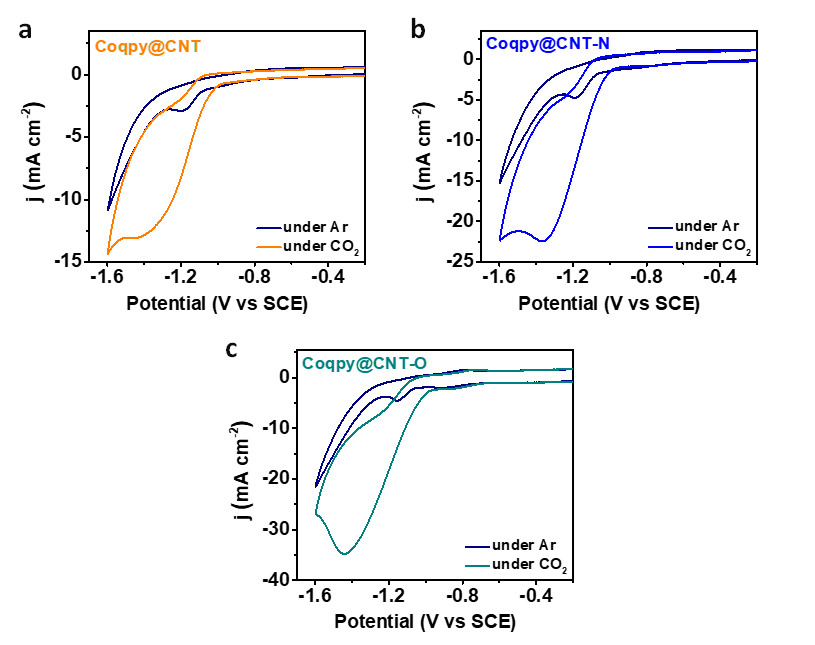
**

**Figure S16.** CV of Coqpy@CNT (a), Coqpy@CNT-N (b), and Coqpy@CNT-O (c) in Ar or CO_2_ saturated 0.5 M KHCO_3_ (*ν* = 100 mV s^-1^).

The CV of Coqpy@CNT in CO_2_-saturated solution shows a much larger catalytic current at a less negative potential of −1.0 V, indicating the preference of the catalyst for CO_2_RR (~223 mV overpotential, E^0^(CO_2_/CO) = −0.11 V *vs.* RHE) over HER.

**
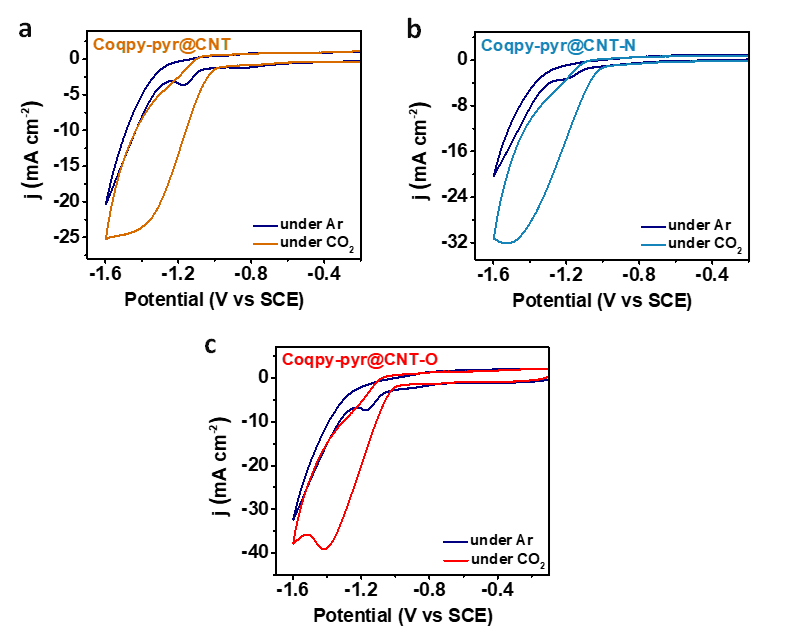
**

**Figure S17.** CV of Coqpy-pyr@CNT (a), Coqpy-pyr@CNT-N (b), and Coqpy-pyr@CNT-O (c) in Ar or CO_2_ saturated 0.5 M KHCO_3_ (*ν* = 100 mV s^-1^).

The increased current densities and obviously positive-shift onset potential after purging CO_2_ into the electrolyte prove the catalytic ability of immobilized Coqpy-pyr for CO_2_RR. The current densities recorded on different MWCNT immobilized Coqpy-pyr follow the sequence: CNT-O > CNT-N > CNT, which is consistent with that of Coqpy.

**
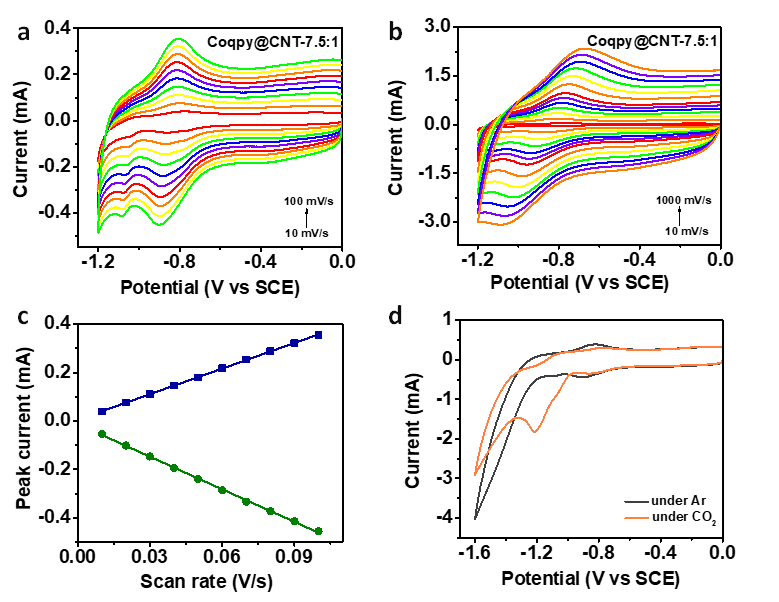
**

**Figure S18.** (a, b) CV of Coqpy@CNT deposited on glassy carbon electrode (Coqpy@CNT@GCE) at various scan rates in Ar-saturated 0.5 M KHCO_3_ (pH 8.8); (c) Evolution of the cathodic and anodic peak current of qpy/qpy^•−^ redox couple as a function of scan rates; (d) CV comparison of Coqpy@CNT@GCE in Ar and CO_2_ saturated 0.5 M KHCO_3_ (*ν* = 100 mV s^-1^).


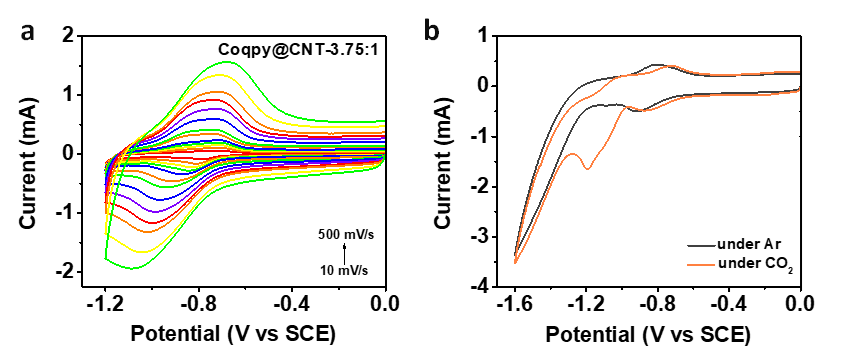


**Figure S19.** (a) CV of Coqpy@CNT@GCE with higher loading (CNT/catalyst mass ratio = 3.75 in ink) at various scan rates in Ar-saturated 0.5 M KHCO_3_ (pH 8.8); (b) The corresponding CV comparison of Coqpy@CNT@GCE in Ar and CO_2_ saturated 0.5 M KHCO_3_ (*ν* = 100 mV s^-1^).

**
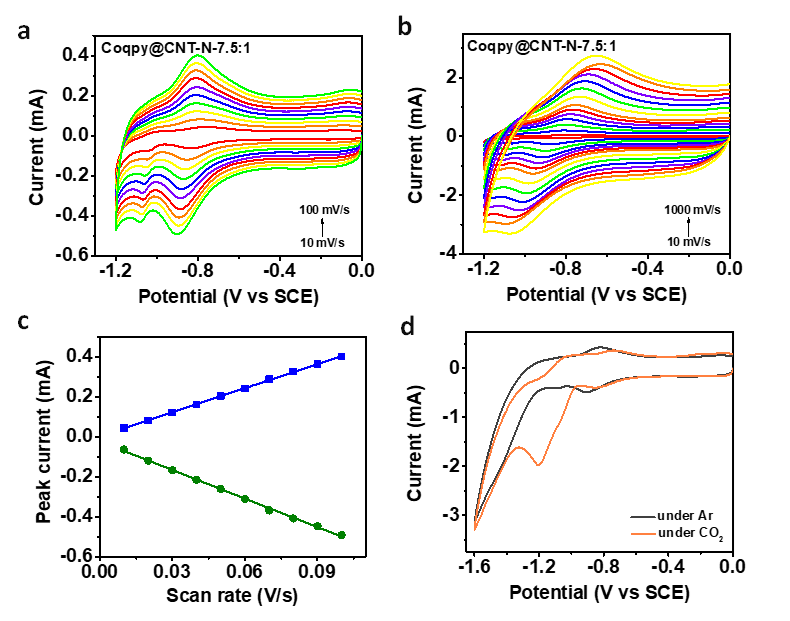
**

**Figure S20.** (a, b) CV of Coqpy@CNT-N@GCE at various scan rates in Ar-saturated 0.5 M KHCO_3_ (pH 8.8); (c) Evolution of the cathodic and anodic peak current of qpy/qpy^•−^ redox couple as a function of scan rates; (d) CV comparison of Coqpy@CNT-N@GCE in Ar and CO_2_ saturated 0.5 M KHCO_3_ (*ν* = 100 mV s^-1^).


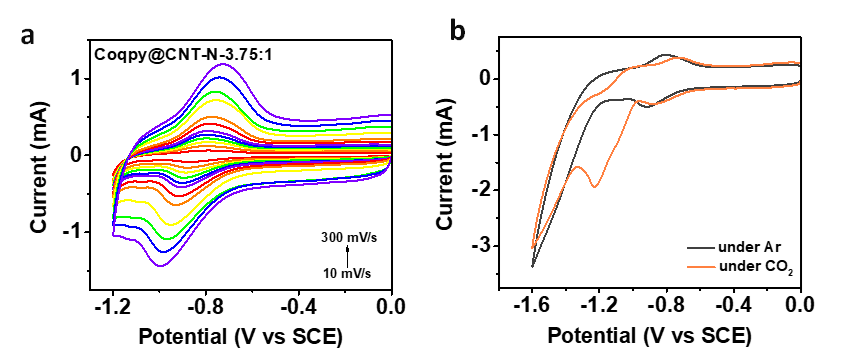


**Figure S21.** (a) CV of Coqpy@CNT-N@GCE with higher loading (CNT/catalyst mass ratio = 3.75 in ink) at various scan rates in Ar-saturated 0.5 M KHCO_3_ (pH 8.8); (b) The corresponding CV comparison of Coqpy@CNT-N@GCE in Ar and CO_2_ saturated 0.5 M KHCO_3_ (*ν* = 100 mV s^-1^).


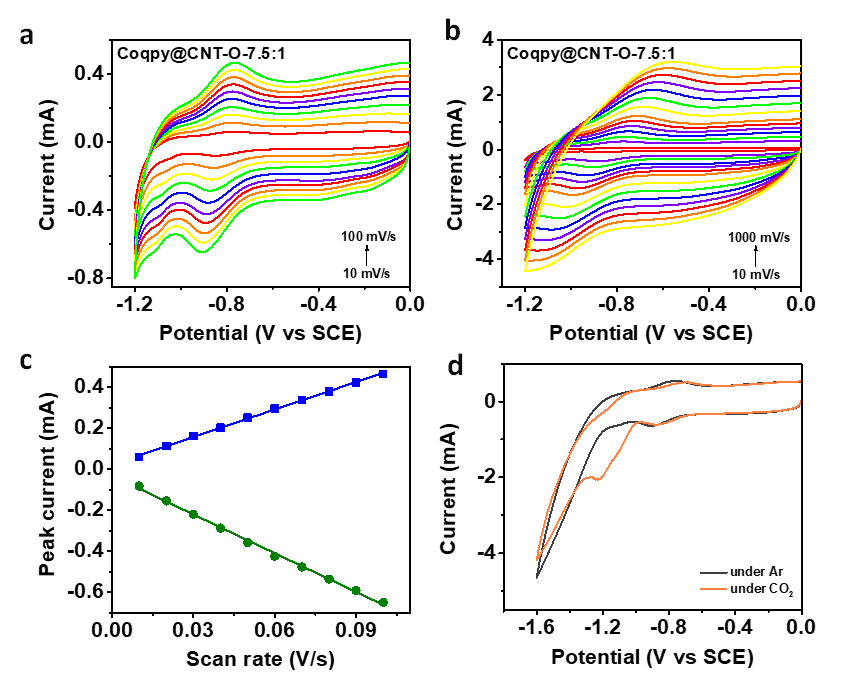


**Figure S22.** (a, b) CV of Coqpy@CNT-O@GCE at various scan rates in Ar-saturated 0.5 M KHCO_3_ (pH 8.8); (c) Evolution of the cathodic and anodic peak current of qpy/qpy^•−^ redox couple as a function of scan rates; (d) CV comparison of Coqpy@CNT-O@GCE in Ar and CO_2_ saturated 0.5 M KHCO_3_ (*ν* = 100 mV s^-1^).

**
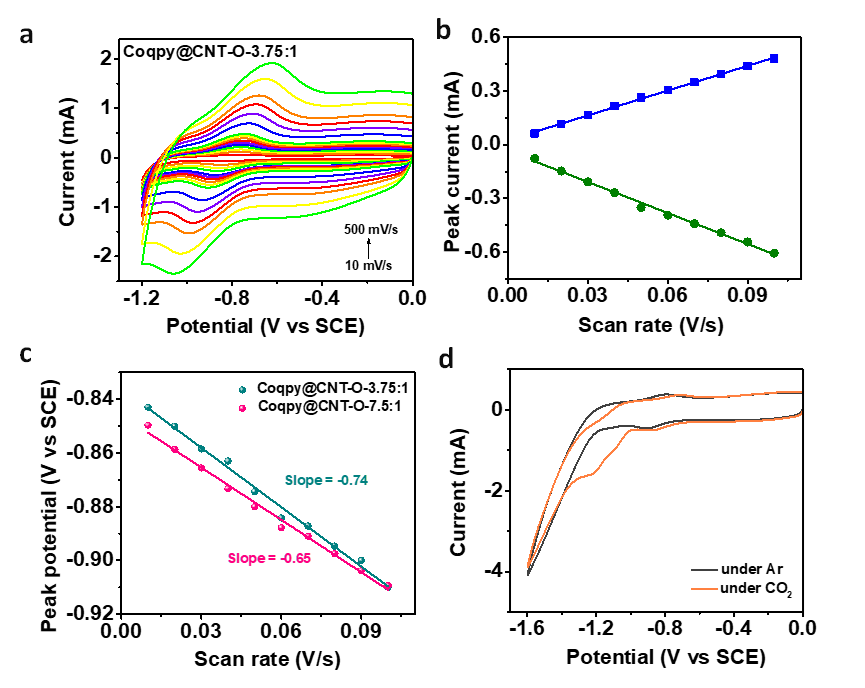
**

**Figure S23.** (a) CV of Coqpy@CNT-O@GCE with higher loading (CNT/catalyst mass ratio = 3.75 in ink) at various scan rates in Ar-saturated 0.5 M KHCO_3_ (pH 8.8); (b) Evolution of the cathodic and anodic peak current of qpy/qpy^•−^ redox couple as a function of scan rates; (c) Variation of redox peak potential with scan rates; (d) The corresponding CV comparison of Coqpy@CNT-O@GCE in Ar and CO_2_ saturated 0.5 M KHCO_3_ (*ν* = 100 mV s^-1^).

**
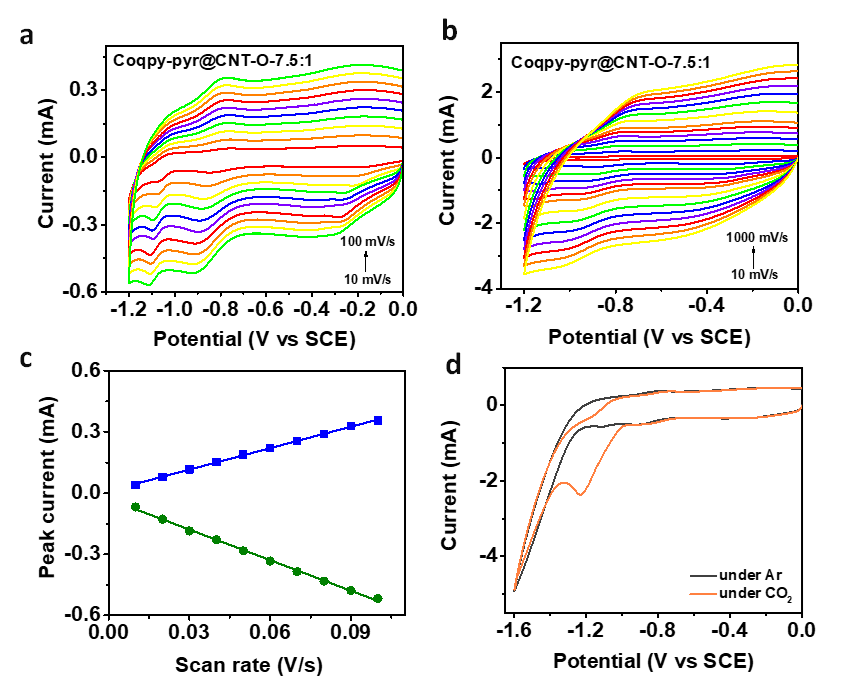
**

**Figure S24.** (a, b) CV of Coqpy-pyr@CNT-O@GCE at various scan rates in Ar-saturated 0.5 M KHCO_3_ (pH 8.8); (c) Evolution of the cathodic and anodic peak current of qpy/qpy^•−^ redox couple as a function of scan rates; (d) CV comparison of Coqpy-pyr@CNT-O@GCE in Ar and CO_2_ saturated 0.5 M KHCO_3_ (*ν* = 100 mV s^-1^).

**
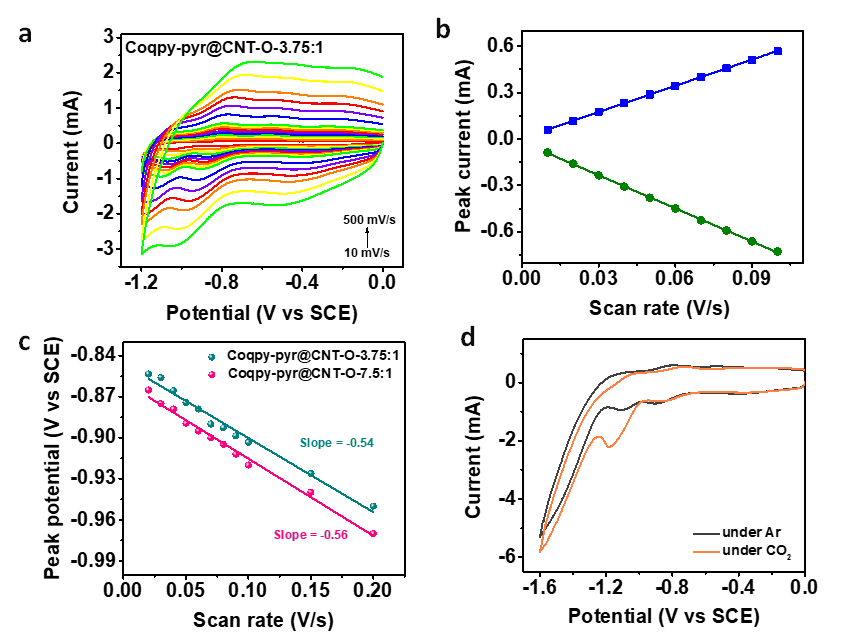
**

**Figure S25.** (a) CV of Coqpy-pyr@CNT-O@GCE with higher loading (CNT/catalyst mass ratio = 3.75 in ink) at various scan rates in Ar-saturated 0.5 M KHCO_3_ (pH 8.8); (b) Evolution of the cathodic and anodic peak current of qpy/qpy^•−^ redox couple as a function of scan rates; (c) Variation of redox peak potential with scan rates; (d) The corresponding CV comparison of Coqpy-pyr@CNT-O@GCE in Ar and CO_2_ saturated 0.5 M KHCO_3_ (*ν* = 100 mV s^-1^).

**Figure S26.** LSV of Coqpy-pyr immobilized on different MWCNT. Conditions: 0.5 M KHCO_3_, SCE reference electrode, scan rate of 100 mV s^-1^.

**Figure S27.** DPV of CNT-O, CNT-N, and CNT in 0.5 M KCl containing 3 mM CoCl_2_.

The higher redox current at around −0.48 V *vs*. SCE corresponding to the Co^II^/Co^I^ couple for CNT-O indicates the superior binding ability of carboxyl for Co sites and better electron transfer between them.

**Figure S28.** Chronoamperometric curves for CO_2_RR over different samples at −1.2 V *vs.* SCE. Conditions: 0.5 M KHCO_3_, SCE reference electrode, pH 7.2.

**Figure S29.** TON_CO_ based on total Co amounts catalyzed by Coqpy immobilized on different MWCNT at −1.2 V *vs*. SCE. Conditions: 0.5 M KHCO_3_, SCE reference electrode, pH 7.2.


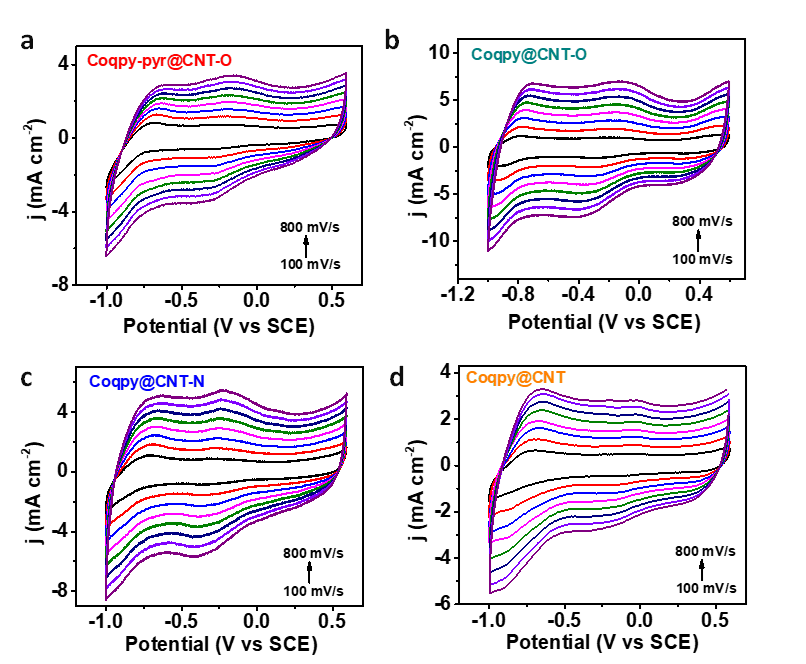


**Figure S30.** Co^II^/Co^I^ redox curves of Coqpy-pyr@CNT-O (a), Coqpy@CNT-O (b), Coqpy@CNT-N (c), and Coqpy@CNT (d) in Ar-saturated 0.5 M KHCO_3_ at different scan rates.

**Figure S31.** H_2_ yield (n_H2_) and FE for different hybrids at −1.2 V *vs*. SCE. Conditions: 0.5 M KHCO_3_, SCE reference electrode, pH 7.2.

For all carbon supports, the FE(H_2_) obtained on immobilized Coqpy-pyr is generally lower than its Coqpy counterpart. The increased H_2_ production on CNT immobilized Coqpy is mainly attributed to its weak binding effect for Co complexes and results in the prevailing hydrogen evolution reaction catalyzed by exposed CNT itself.

**Figure S32.** Comparison of CV of Coqpy-pyr@CNT-O and Coqpy@CNT-O under CO_2_ atmosphere (0.5 M KHCO_3_, *ν* = 100 mV s^-1^).

Though with lower molecular loading, the current density recorded on Coqpy-pyr@CNT-O is larger than that of Coqpy@CNT-O, demonstrating its superior catalytic activity for CO_2_RR.

**Figure S33.** TON_CO_ based on total Co quantities catalyzed by Coqpy-pyr@CNT-O and Coqpy@CNT-O at different potentials. Conditions: 0.5 M KHCO_3_, SCE reference electrode, pH 7.2.

**Figure S34.** FE of CO and H_2_ over Coqpy-pyr@CNT-O at different current densities.

Coqpy-pyr@CNT-O maintains a high CO FE of ~92% at current densities of 100 and 150 mA cm^-^², but decreases to 80.9% at 200 mA cm^-^² due to the flooding and promoted H_2_ production.

**Figure S35.** CV of Coqpy-pyr@CNT-O and Coqpy@CNT-O under Ar before and after CPE test at −1.2 V *vs*. SCE. Conditions: 0.5 M KHCO_3_, SCE reference electrode, scan rate of 100 mV s^-1^.

The nearly unchanged CV of Coqpy-pyr@CNT-O proves its high electrochemical stability while the attenuated redox behavior for Coqpy@CNT-O implies that Coqpy molecules are detached from the electrode surface.

**
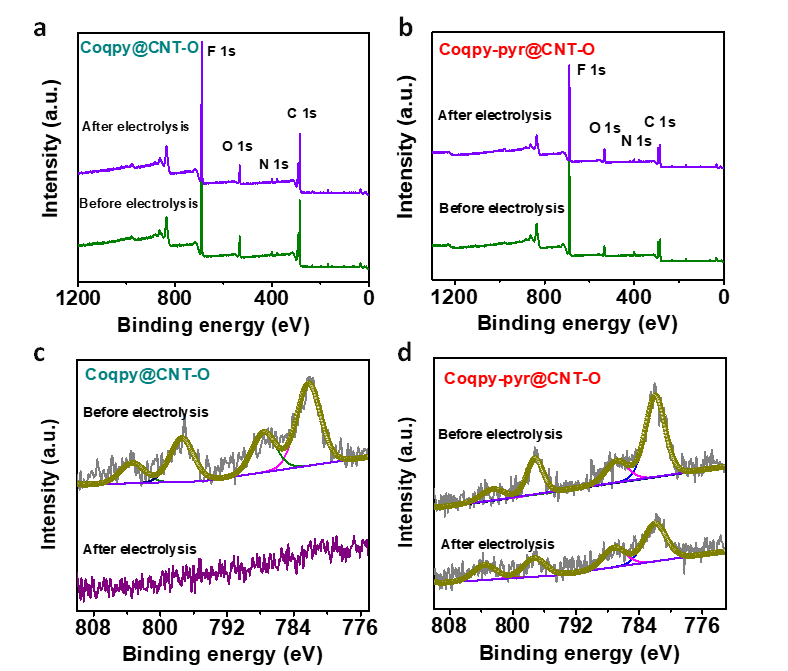
**

**Figure S36.** Survey and Co 2p XPS scans of Coqpy@CNT-O (a, c) and Coqpy-pyr@CNT-O (b, d) before and after CPE at –1.2 V *vs*. SCE.


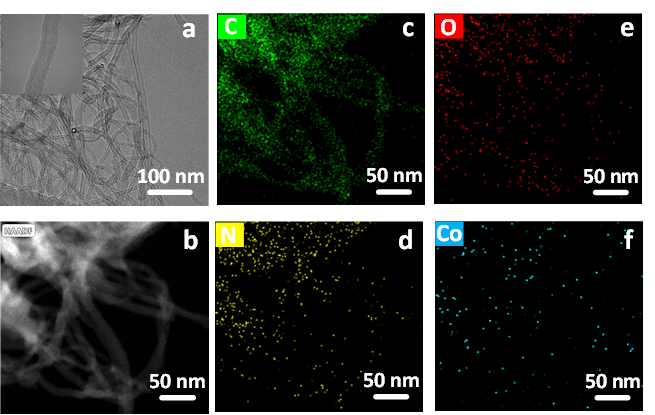


**Figure S37.** High-magnification TEM image (a), HAADF-STEM image and corresponding EDS elemental mappings (b-f) of Coqpy-pyr@CNT-O after CPE at –1.2 V *vs*. SCE.

**Figure S38.** Long-term stability and corresponding CO and H_2_ FE of Coqpy-pyr@CNT-O at a constant current density of 100 mA cm^-^² using a flow cell.

**Figure S39.** Co 2p XPS scan of Coqpy-pyr@CNT-O after electrolysis at 100 mA cm^-^².


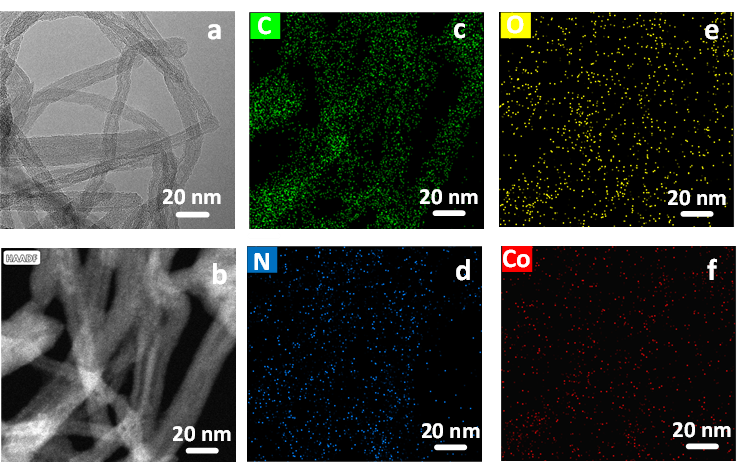


**Figure S40.** High-magnification TEM image (a), HAADF-STEM image and corresponding EDS elemental mappings (b-f) of Coqpy-pyr@CNT-O after electrolysis at 100 mA cm^-^².

**Figure S41.** Tafel plot of Coqpy-pyr@CNT-O and the corresponding CPE curves. Conditions: 0.5 M KHCO_3_, SCE reference electrode, pH 7.2.

**Figure S42.** LSV of CFP, CNT, CNT-N, and CNT-O in Ar or CO_2_ saturated 0.5 M KHCO_3_ (*ν* = 100 mV s^-1^).

The catalytic currents of blank MWCNT are primarily ascribed to the hydrogen evolution reaction, while the enhanced current densities under CO_2_ are caused by the reduced pH of the electrolyte.

**Figure S43.** Comparison of LSV of Coqpy-pyr and Coqpy directly deposited on CFP in Ar or CO_2_ saturated 0.5 M KHCO_3_ (*ν* = 100 mV s^-1^).

Coqpy-pyr supported on CFP exhibits higher current density than that of Coqpy under a CO_2_ atmosphere, implying its higher catalytic activity for CO_2_RR. This performance improvement is ascribed to its stronger π-π interaction with CFP induced by pyrene groups, leading to accelerated electron migration.

**Figure S44.** Chronoamperometric curves of CFP, different MWCNT, Coqpy-pyr and Coqpy on CFP over 2 h-CPE at −1.2 V *vs*. SCE. Conditions: 0.5 M KHCO_3_, SCE reference electrode, pH 7.2.

The insignificant current densities of blank MWCNT for CO_2_RR compared with that of Coqpy-pyr and Coqpy verify the main catalytic active species being from Co complexes. Coqpy-pyr and Coqpy on CFP display sharply decreased current responses relative to MWCNT-supported ones, indicating the pivotal role of MWCNT in boosting the CO_2_RR activity of Co complexes.

**Figure S45.** ^13^C isotope labeling experiments of Coqpy-pyr@CNT-O conducted in 0.5 M KCl.

**Figure S46.** Catalytic current (a) and charge (b) versus time plots of CO_2_RR catalyzed by 0.5 mM Coqpy and Coqpy-pyr at −1.3 V *vs*. SCE in 0.1 M [^n^Bu_4_N]PF_6_ DMF solution.

**Figure S47.** Optimized geometry of adsorbed intermediates on Coqpy@CNT (C: brown; N: pale blue; O: red; H: white; Co: navy).

**Table S1.** Cl/Co atomic ratio of Coqpy-pyr before and after immobilization on CNT-O from XPS analysis.

| **Samples** | **Cl atomic percentage (%)** | **Co atomic percentage**  **(%)** | **Cl/Co atomic ratio** |
| --- | --- | --- | --- |
| Coqpy-pyr | 1.78 | 0.89 | 2.0 |
| Coqpy-pyr@CNT-O | 0.31 | 0.31 | 1.0 |

**Table S2.** Cobalt contents of immobilized samples on electrode determined by ICP-MS.

| **Samples** | **Cobalt content (nmol cm^-2^)** |
| --- | --- |
| Coqpy-pyr@CNT-O | 8.2 |
| Coqpy@CNT | 27.3 |
| Coqpy@CNT-N | 26.5 |
| Coqpy@CNT-O | 17.1 |

**Table S3.** Performance comparison of Coqpy-pyr@CNT-O with previously reported immobilized molecular catalysts for CO_2_RR.

| **Catalyst** | | **Electrolyte** | **Potential**  **(V *vs.* RHE)** | **FE(CO) (%)** | **TOF**  **(s^-1^)** | **Ref.** |
| --- | --- | --- | --- | --- | --- | --- |
| Coqpy-pyr@CNT-O | | 0.5 M KHCO_3_ | −0.63 | 97.7 | 79^a^  7.4^b^ | This work |
| Coqpy/CNTs | 0.5 M NaHCO_3_ | | −0.58 | 99 | 12.0^a^ | ^[^[^14^](#_ENREF_14)^]^ |
| Co-qpyCOOH/CNT | 0.1 M NaHCO_3_ | | −0.75 | ~100 | 1.4^b^ | ^[^[^15^](#_ENREF_15)^]^ |
| CoPc_2_@MWCNTs | 0.5 M NaHCO_3_ | | −0.676 | 93 | 6.8^b^ | ^[^[^16^](#_ENREF_16)^]^ |
| N_3_NiPc-CNT | 0.5 M KHCO_3_ | | −0.93 | 100 | 9.0^b^ | ^[^[^17^](#_ENREF_17)^]^ |
| CoPc/GDY/G | 0.1 M KHCO_3_ | | −0.70 | 96 | 9.1^b^ | ^[^[^18^](#_ENREF_18)^]^ |
| CoPc-H_2_Pc | 0.5 M KHCO_3_ | | −0.65 | 96 | 27.1^bc^ | ^[^[^19^](#_ENREF_19)^]^ |
| CCG/CoPc-A | 0.1 M KHCO_3_ | | −0.69 | 90 | ~6.0^a^ | ^[^[^20^](#_ENREF_20)^]^ |
| CoTMAPc@CNT | 0.5 M KHCO_3_ | | −0.62 | ~97 | 102.9^a^ | ^[^[^21^](#_ENREF_21)^]^ |
| CoPc-PDQ-COF | 0.5 M KHCO_3_ | | −0.66 | 96 | 3.2^b^ | ^[^[^22^](#_ENREF_22)^]^ |
| CoPP@CNT | 0.5 M NaHCO_3_ | | −0.65 | 90 | 2.1^b^ | ^[^[^23^](#_ENREF_23)^]^ |
| CoPc/CNT-MD | 0.5 M KHCO_3_ | | −1.0 | ~97 | 13.0^b^ | ^[^[^24^](#_ENREF_24)^]^ |
| CoPc-CN/CNT | 0.1 M KHCO_3_ | | −0.63 | 98 | 4.1^b^ | ^[^[^25^](#_ENREF_25)^]^ |
| STPyP-Co | 0.5 M KHCO_3_ | | −0.62 | ~96 | 4.2^a^ | ^[^[^26^](#_ENREF_26)^]^ |
| SCo-G | 0.1 M KHCO_3_ | | −0.87 | ~90 | 2.4^b^ | ^[^[^27^](#_ENREF_27)^]^ |
| CoPc@HCS-6 | 0.5 M KHCO_3_ | | −0.82 | 96 | 24.2^b^ | ^[^[^28^](#_ENREF_28)^]^ |
| CoTAP-cov | 0.5 M KHCO_3_ | | −0.67 | ~100 | 6.0^b^ | ^[^[^29^](#_ENREF_29)^]^ |
| CoN_4_H-Ph_Pyr_ \|MWCNT | 0.5 M KHCO_3_ | | –0.56 | 94 | 2.8^a^ | ^[^[^30^](#_ENREF_30)^]^ |
| MnBpy  \|MWCNT | 0.5 M KHCO_3_ | | –0.76 | 72 | - | ^[^[^31^](#_ENREF_31)^]^ |
| EtO_8_−CoPc\|CNP | 1.0 M KHCO_3_ | | - | ~95 | 187.3^ac^ | ^[^[^32^](#_ENREF_32)^]^ |

A and b refer to TOF values derived from the CO generated per molecular complex based on electrocatalytic active amount and total metal loading determined by ICP-MS analysis per unit time, respectively; C refers to the electrolysis in a flow cell while the others are performed in H-type cell or single-compartment cell.

**Table S4.** Co atomic percentage of Coqpy@CNT-O and Coqpy-pyr@CNT-O determined by XPS before and after catalysis at −1.2 V *vs*. SCE.

| **Samples** | **Co atomic percentage before catalysis (%)** | **Co atomic percentage**  **after catalysis (%)** | **Co loss percentage (%)** |
| --- | --- | --- | --- |
| Coqpy@CNT-O | 0.38 | 0.13 | 65.8 |
| Coqpy-pyr@CNT-O | 0.31 | 0.28 | 9.7 |

**References**

[1] Z. Guo, G. Chen, C. Cometto, B. Ma, H. Zhao, T. Groizard, L. Chen, H. Fan, W.-L. Man, S.-M. Yiu, K.-C. Lau, T.-C. Lau, M. Robert, Selectivity control of CO versus HCOO^−^ production in the visible-light-driven catalytic reduction of CO_2_ with two cooperative metal sites. *Nat. Catal.* **2019**, *2*, 801-808.

[2] B. Ma, M. Blanco, L. Calvillo, L. Chen, G. Chen, T.-C. Lau, G. Dražić, J. Bonin, M. Robert, G. Granozzi, Hybridization of molecular and graphene materials for CO_2_ photocatalytic reduction with selectivity control. *J. Am. Chem. Soc.* **2021**, *143*, 8414-8425.

[3] B. Ravel, M. Newville, M. ATHENA, ARTEMIS, HEPHAESTUS: data analysis for X-ray absorption spectroscopy using IFEFFIT. *J. Synchrotron Rad.* **2005**, *12*, 537-541.

[4] H. Funke, A. C. Scheinost, M. Chukalina, Wavelet analysis of extended x-ray absorption fine structure data. *Phys. Rev. B* **2005**, *71*, 094110.

[5] H. Funke, M. Chukalina, A. C. Scheinost, A new FEFF-based wavelet for EXAFS data analysis. *J. Synchrotron Rad.* **2007**, *14*, 426-432.

[6] G. Kresse, J. Furthmüller, Efficient iterative schemes for ab initio total-energy calculations using a plane-wave basis set. *Phys. Rev. B* **1996**, *54*, 11169-11186.

[7] G. Kresse, J. Furthmüller, Efficiency of ab-initio total energy calculations for metals and semiconductors using a plane-wave basis set. *Comp. Mater. Sci.* **1996**, *6*, 15-50.

[8] G. Kresse, D. Joubert, From ultrasoft pseudopotentials to the projector augmented-wave method. *Phys. Rev. B* **1999**, *59*, 1758-1775.

[9] P. E. Blöchl, Projector augmented-wave method. *Phys. Rev. B* **1994**, *50*, 17953-17979.

[10] J. P. Perdew, K. Burke, M. Ernzerhof, Generalized gradient approximation made simple. *Phys. Rev. Lett.* **1996**, *77*, 3865-3868.

[11] B. Hammer, L. B. Hansen, J. K. Nørskov, Improved adsorption energetics within density-functional theory using revised Perdew-Burke-Ernzerhof functionals. *Phys. Rev. B* **1999**, *59*, 7413-7421.

[12] R. Nelson, C. Ertural, J. George, V. L. Deringer, G. Hautier, R. Dronskowski, LOBSTER: Local orbital projections, atomic charges, and chemical-bonding analysis from projector-augmented-wave-based density-functional theory. *J. Comput. Chem.* **2020**, *41*, 1931-1940.

[13] J. K. Nørskov, J. Rossmeisl, A. Logadottir, L. Lindqvist, J. R. Kitchin, T. Bligaard, H. Jónsson, Origin of the overpotential for oxygen reduction at a fuel-cell cathode. *J. Phys. Chem. B* **2004**, *108*, 17886-17892.

[14] M. Wang, L. Chen, T.-C. Lau, M. Robert, A hybrid Co quaterpyridine complex/carbon nanotube catalytic material for CO_2_ reduction in water. *Angew. Chem. Int. Ed.* **2018**, *57*, 7769-7773.

[15] V. Reddu, L. Sun, X. Li, H. Jin, S. Wang, X. Wang, Highly selective and efficient electroreduction of CO_2_ in water by quaterpyridine derivative-based molecular catalyst noncovalently tethered to carbon nanotubes. *SmartMat* **2022**, *3*, 151-162.

[16] M. Wang, K. Torbensen, D. Salvatore, S. Ren, D. Joulié, F. Dumoulin, D. Mendoza, B. Lassalle-Kaiser, U. Işci, C. P. Berlinguette, M. Robert, CO_2_ electrochemical catalytic reduction with a highly active cobalt phthalocyanine. *Nat. Commun.* **2019**, *10*, 3602.

[17] D.-D. Ma, S.-G. Han, C. Cao, W. Wei, X. Li, B. Chen, X.-T. Wu, Q.-L. Zhu, Bifunctional single-molecular heterojunction enables completely selective CO_2_-to-CO conversion integrated with oxidative 3D nano-polymerization. *Energy Environ. Sci.* **2021**, *14*, 1544-1552.

[18] H. Gu, L. Zhong, G. Shi, J. Li, K. Yu, J. Li, S. Zhang, C. Zhu, S. Chen, C. Yang, Y. Kong, C. Chen, S. Li, J. Zhang, L. Zhang, Graphdiyne/graphene heterostructure: A universal 2D scaffold anchoring monodispersed transition-metal phthalocyanines for selective and durable CO_2_ electroreduction. *J. Am. Chem. Soc.* **2021**, *143*, 8679-8688.

[19] R. Wang, X. Wang, W. Weng, Y. Yao, P. Kidkhunthod, C. Wang, Y. Hou, J. Guo, Proton/electron donors enhancing electrocatalytic activity of supported conjugated microporous polymers for CO_2_ reduction. *Angew. Chem. Int. Ed.* **2022**, *61*, e202115503.

[20] J. Choi, P. Wagner, S. Gambhir, R. Jalili, D. R. MacFarlane, G. G. Wallace, D. L. Officer, Steric modification of a cobalt phthalocyanine/graphene catalyst to give enhanced and stable electrochemical CO_2_ reduction to CO. *ACS Energy Lett.* **2019**, *4*, 666-672.

[21] J. Su, J.-J. Zhang, J. Chen, Y. Song, L. Huang, M. Zhu, B. I. Yakobson, B. Z. Tang, R. Ye, Building a stable cationic molecule/electrode interface for highly efficient and durable CO_2_ reduction at an industrially relevant current. *Energy Environ. Sci.* **2021**, *14*, 483-492.

[22] N. Huang, K. H. Lee, Y. Yue, X. Xu, S. Irle, Q. Jiang, D. Jiang, A stable and conductive metallophthalocyanine framework for electrocatalytic carbon dioxide reduction in water. *Angew. Chem. Int. Ed.* **2020**, *59*, 16587-16593.

[23] M. Zhu, J. Chen, L. Huang, R. Ye, J. Xu, Y.-F. Han, Covalently grafting cobalt porphyrin onto carbon nanotubes for efficient CO_2_ electroreduction. *Angew. Chem. Int. Ed.* **2019**, *58*, 6595-6599.

[24] X. Wu, J. W. Sun, P. F. Liu, J. Y. Zhao, Y. Liu, L. Guo, S. Dai, H. G. Yang, H. Zhao, Molecularly dispersed cobalt phthalocyanine mediates selective and durable CO_2_ reduction in a membrane flow cell. *Adv. Funct. Mater.* **2022**, *32*, 2107301.

[25] X. Zhang, Z. Wu, X. Zhang, L. Li, Y. Li, H. Xu, X. Li, X. Yu, Z. Zhang, Y. Liang, H. Wang, Highly selective and active CO_2_ reduction electrocatalysts based on cobalt phthalocyanine/carbon nanotube hybrid structures. *Nat. Commun.* **2017**, *8*, 14675.

[26] J. Han, P. An, S. Liu, X. Zhang, D. Wang, Y. Yuan, J. Guo, X. Qiu, K. Hou, L. Shi, Y. Zhang, S. Zhao, C. Long, Z. Tang, Reordering d orbital energies of single-site catalysts for CO_2_ electroreduction. *Angew. Chem. Int. Ed.* **2019**, *58*, 12711-12716.

[27] J. Wang, X. Huang, S. Xi, H. Xu, X. Wang, Axial modification of cobalt complexes on heterogeneous surface with enhanced electron transfer for carbon dioxide reduction. *Angew. Chem. Int. Ed.* **2020**, *59*, 19162-19167.

[28] S. Gong, W. Wang, X. Xiao, J. Liu, C. Wu, X. Lv, Elucidating influence of the existence formation of anchored cobalt phthalocyanine on electrocatalytic CO_2_-to-CO conversion. *Nano Energy* **2021**, *84*, 105904.

[29] S. Gu, A. N. Marianov, Y. Jiang, Covalent grafting of cobalt aminoporphyrin-based electrocatalyst onto carbon nanotubes for excellent activity in CO_2_ reduction. *Appl. Catal. B-Environ.* **2022**, *300*, 120750.

[30] M. Haake, D. Aldakov, J. Pérard, G. Veronesi, A. A. Tapia, B. Reuillard, V. Artero, Impact of the surface microenvironment on the redox properties of a Co-based molecular cathode for selective aqueous electrochemical CO_2_-to-CO reduction. *J. Am. Chem. Soc.* **2024**, *146*, 15345-15355.

[31] E. E. DeLuca, T. Chan, J. M. Taylor, B. Lee, R. R. Prabhakar, C. P. Kubiak, Steric effects on CO_2_ reduction with substituted Mn(bpy)(CO)_3_X-type catalysts on multiwalled carbon nanotubes reveal critical mechanistic details. *ACS Catal.* **2024**, *14*, 2071-2083.

[32] S. Ren, E. W. Lees, C. Hunt, A. Jewlal, Y. Kim, Z. Zhang, B. A. W. Mowbray, A. G. Fink, L. Melo, E. R. Grant, C. P. Berlinguette, Catalyst aggregation matters for immobilized molecular CO_2_RR electrocatalysts. *J. Am. Chem. Soc.* **2023**, *145*, 4414-4420.
